# Supplementary material for: The pink salmon genome: Uncovering the genomic consequences of a two-year life cycle
Source: PLoS One. 2021 Dec 17;16(12):e0255752. doi: 10.1371/journal.pone.0255752 (PMC8682878; doi:10.1371/journal.pone.0255752)
Supplement: S1 Fig — A chromosome-by-chromosome comparison of the odd and even-year genome assemblies. Each slide has two figures shown side-by-side with the odd-year scaffolds aligned to the corresponding odd-year chromosome on the left and the even-year scaffolds aligned to the corresponding odd-year chromosome. CHROMEISTER [111] was used to align the scaffolds to the chromosomes. On the y-axes, the scaffold number (in descending order from the top) is shown, with dashed lines delineating the scaffold alignments. The chromosome position is shown on the x-axes. The y-axes are not equivalent between figures, but the x-axes are. (PDF) [file pone.0255752.s002.pdf]

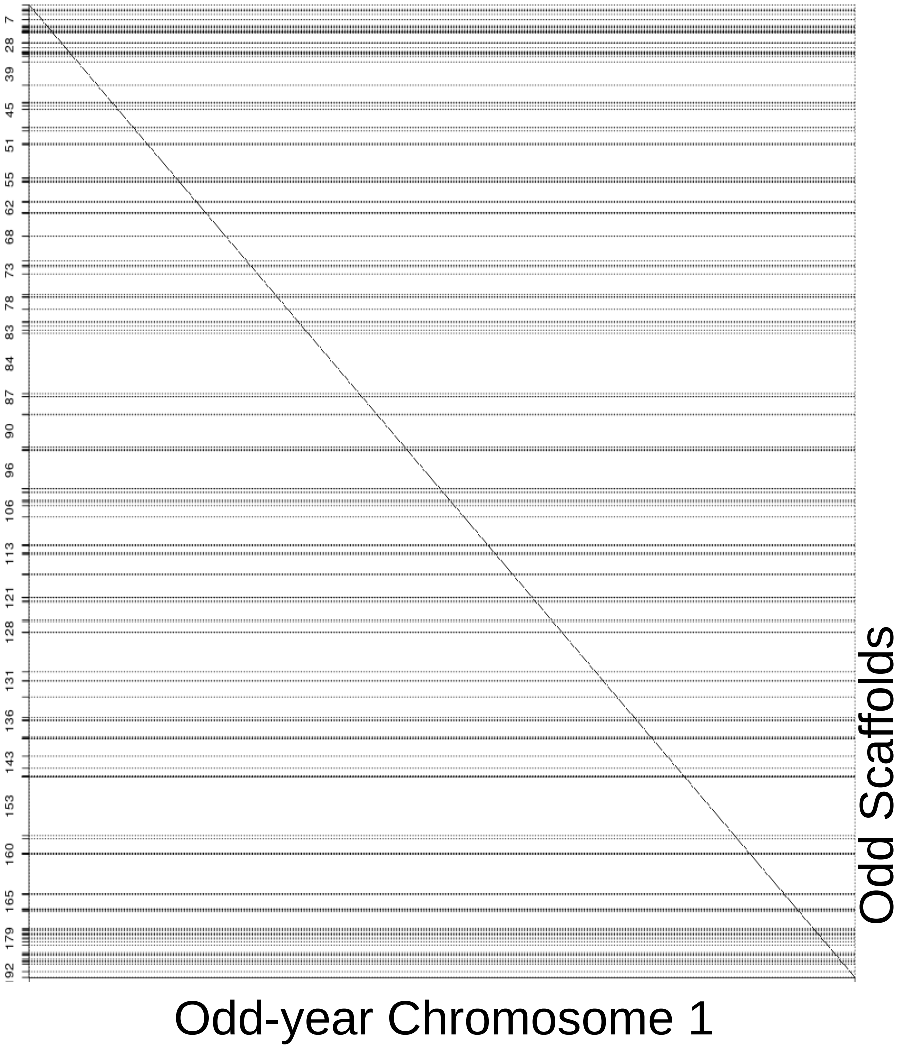
7
28
39
45
51
55
62
68
73
78
83
84
87
90
96
106
106
113
121
128
131
136
143
153
160
165
179
192
Odd-year Chromosome 1
Odd Scaffolds

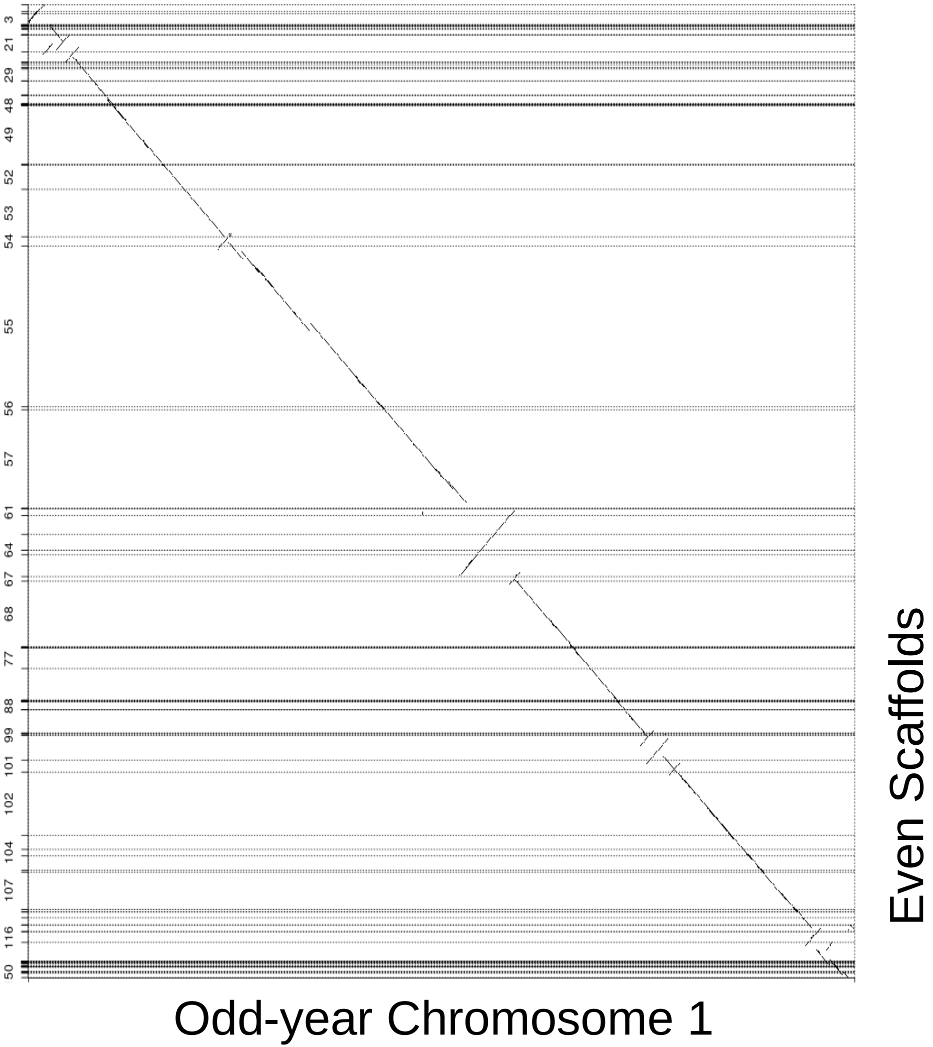
3
21
29
48
49
52
53
54
55
56
57
61
64
67
68
77
88
99
101
102
104
107
116
50
Odd-year Chromosome 1
Even Scaffolds

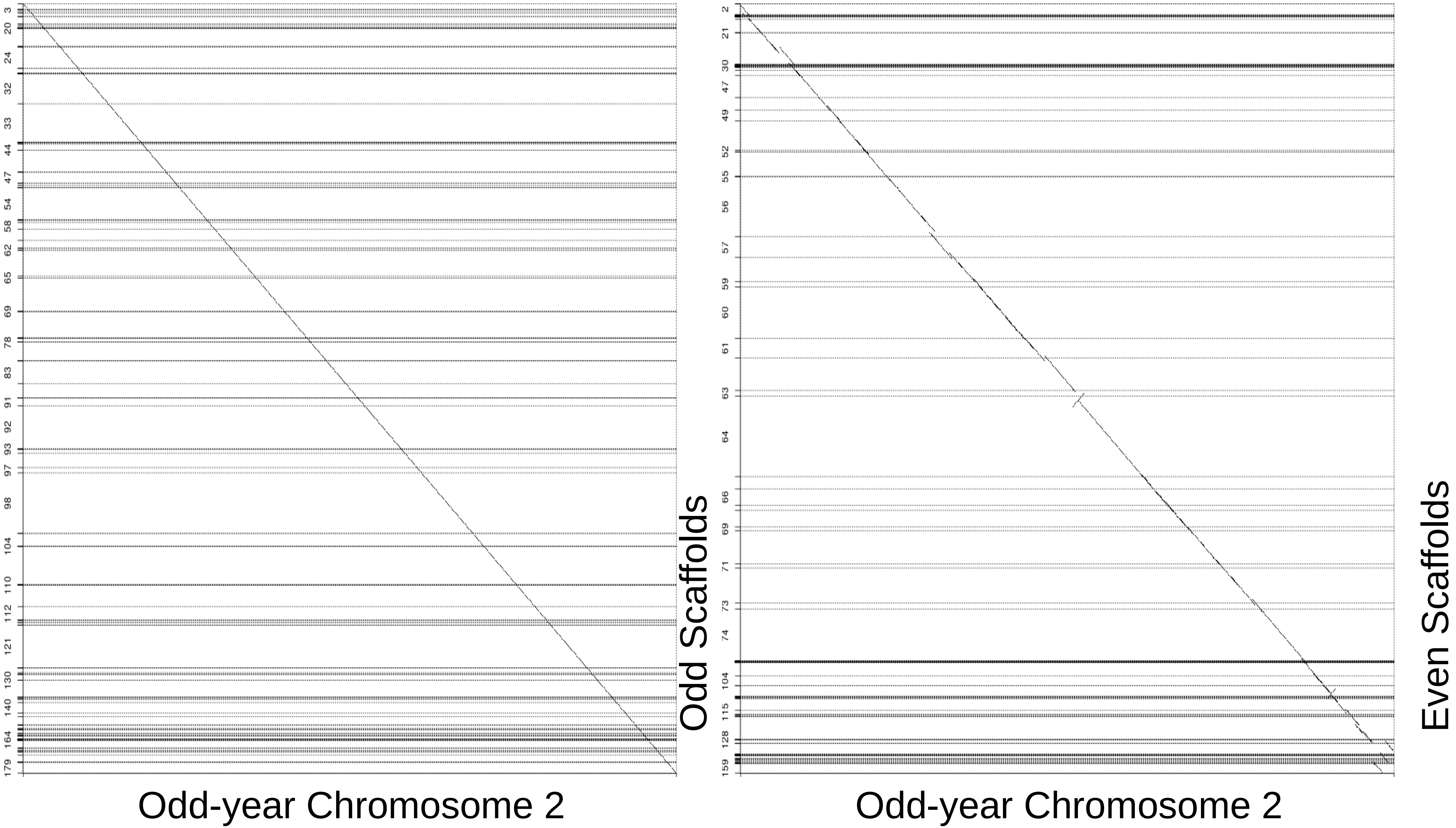
Odd Scaffolds
Odd-year Chromosome 2
Even Scaffolds
Odd-year Chromosome 2

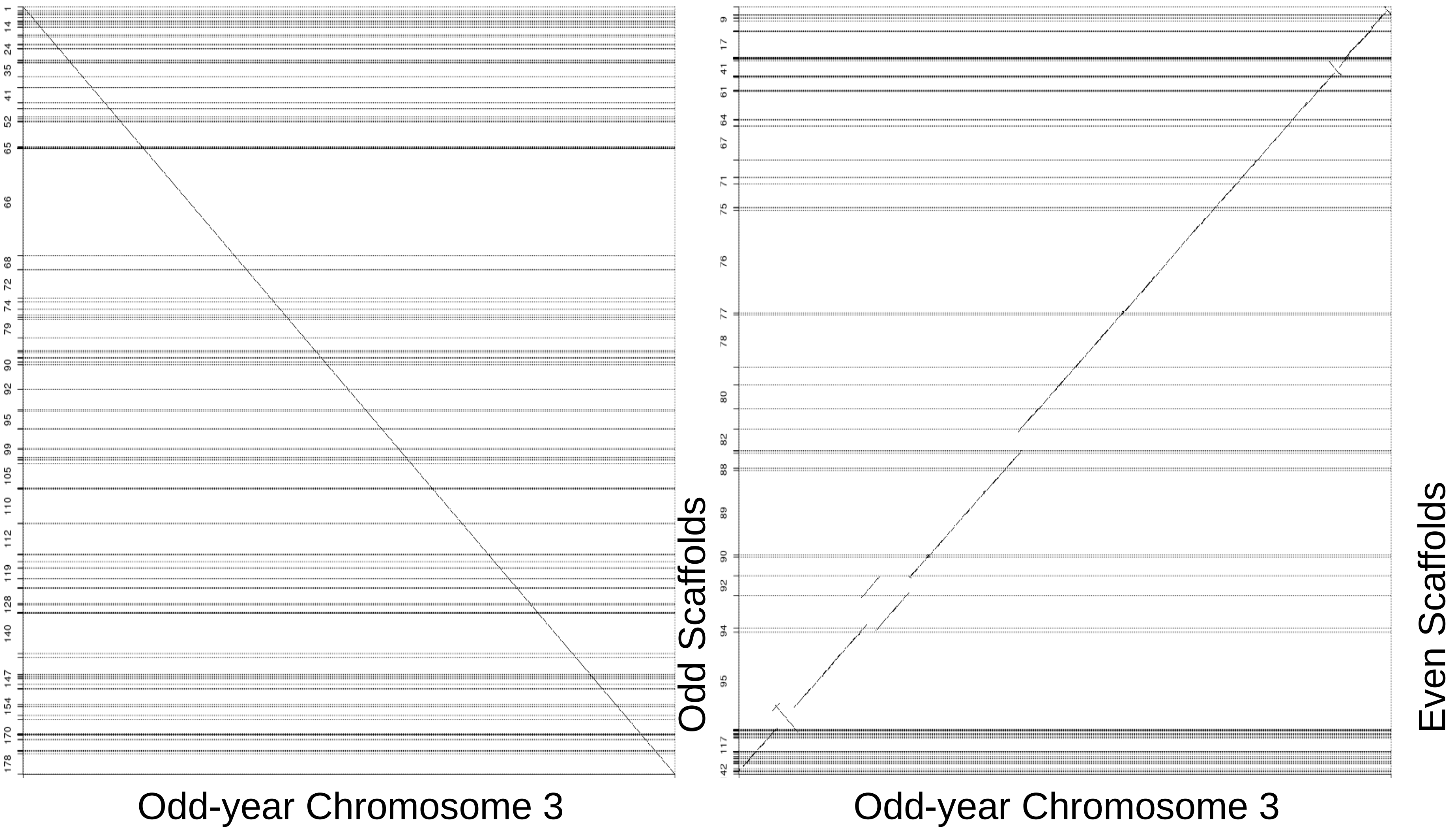
Odd-year Chromosome 3
Odd Scaffolds
Odd-year Chromosome 3
Even Scaffolds

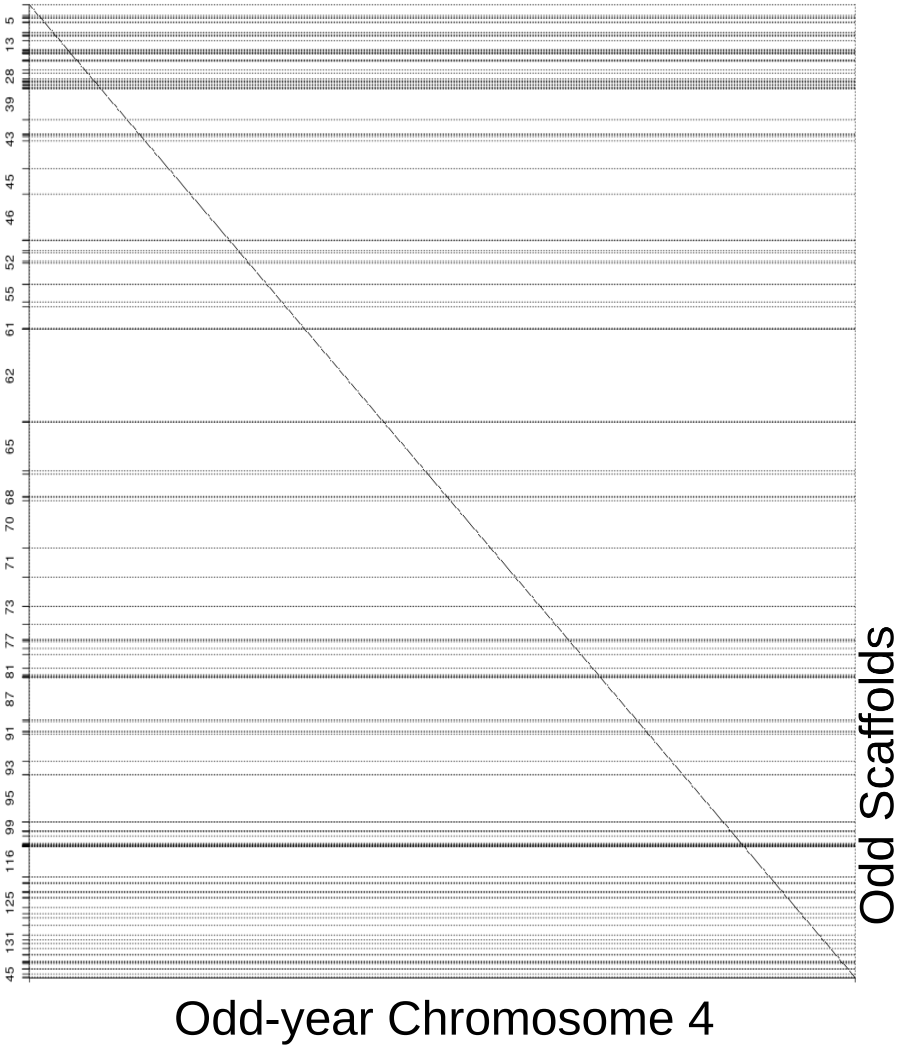
5
13
28
28
39
43
45
46
52
55
61
62
65
68
70
71
73
77
81
87
91
93
95
99
116
125
131
45
Odd Scaffolds
Odd-year Chromosome 4

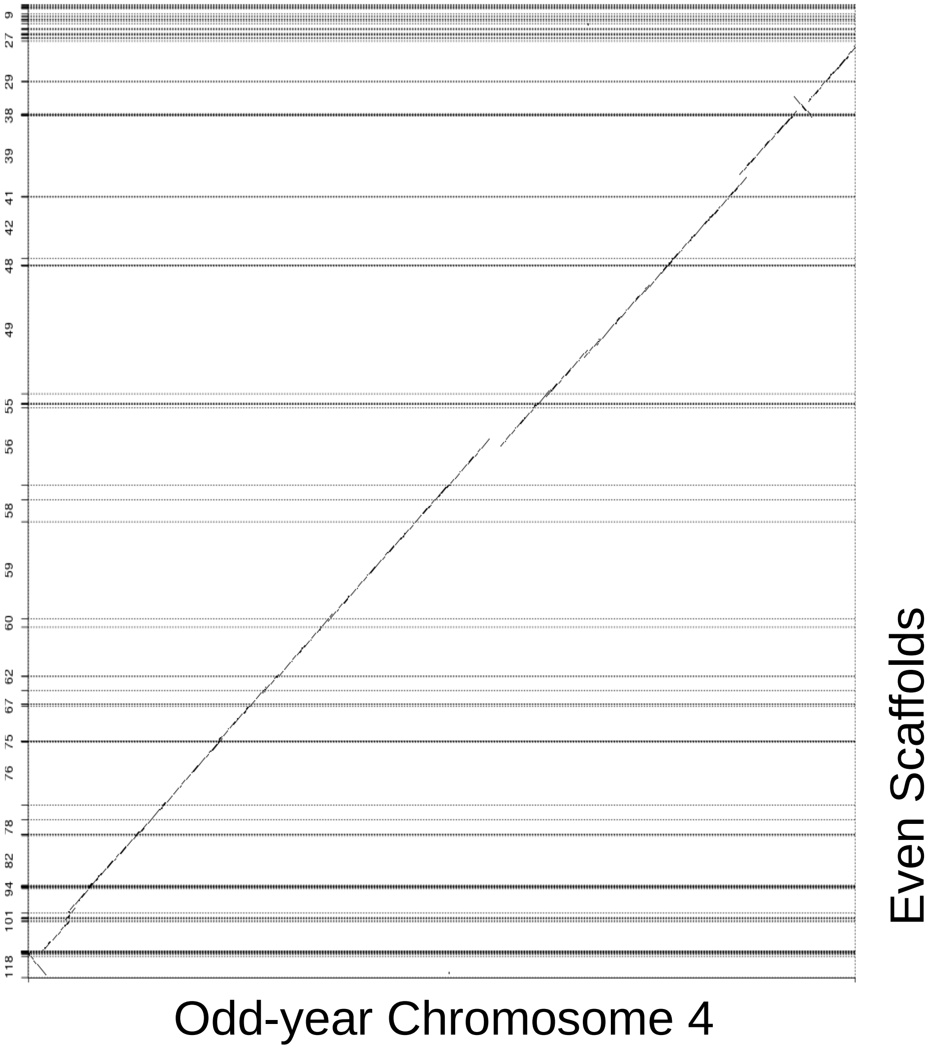
9
27
29
38
39
41
42
48
49
55
56
58
59
60
62
67
75
76
78
82
94
101
118
Even Scaffolds
Odd-year Chromosome 4

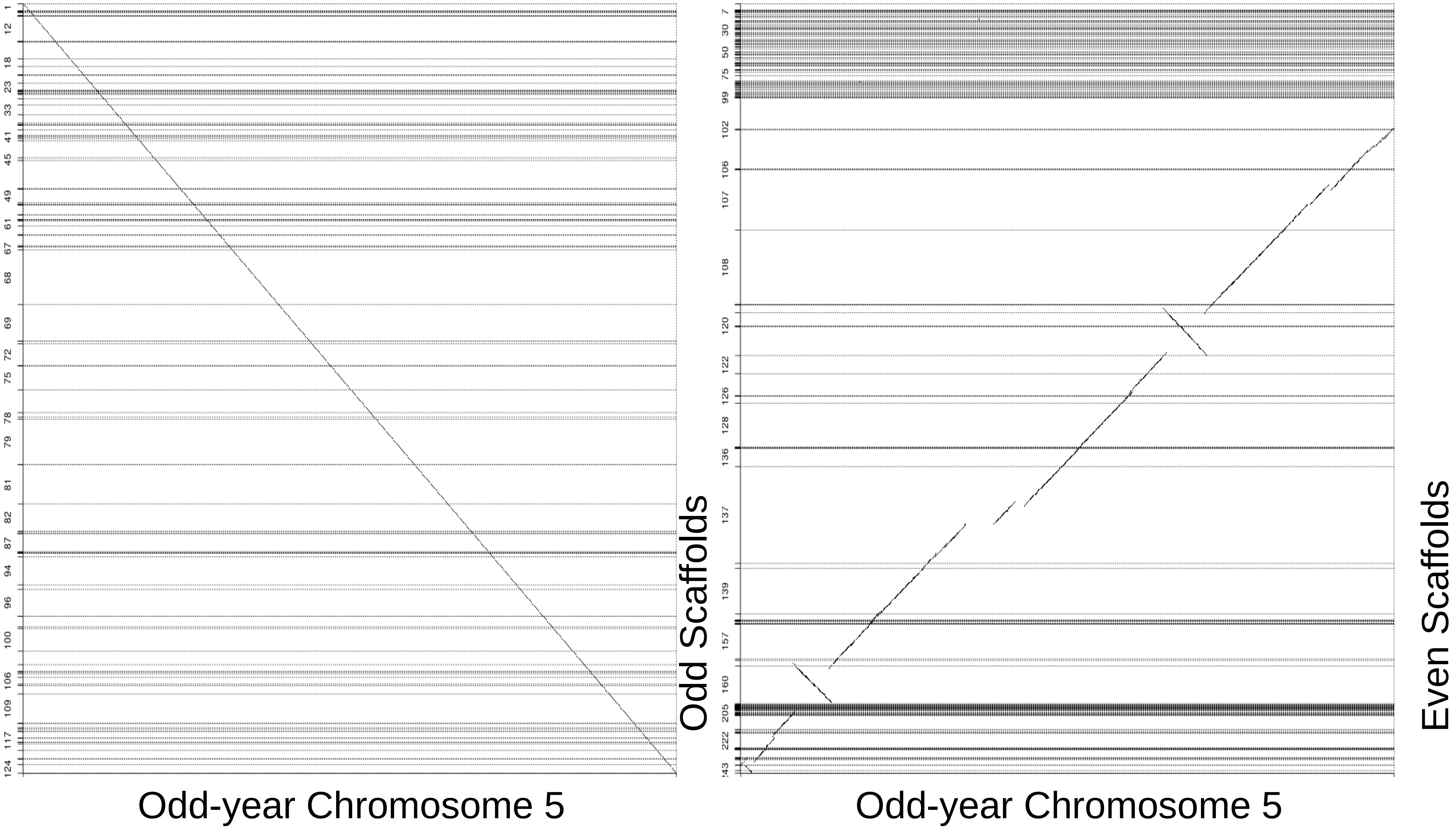
1
12
18
23
33
41
45
49
61
67
68
69
72
75
78
79
81
82
87
94
96
100
106
109
117
124
Odd-year Chromosome 5
Odd Scaffolds
7
30
50
75
99
102
106
107
108
120
122
126
128
136
137
139
157
160
205
222
243
Odd-year Chromosome 5
Even Scaffolds

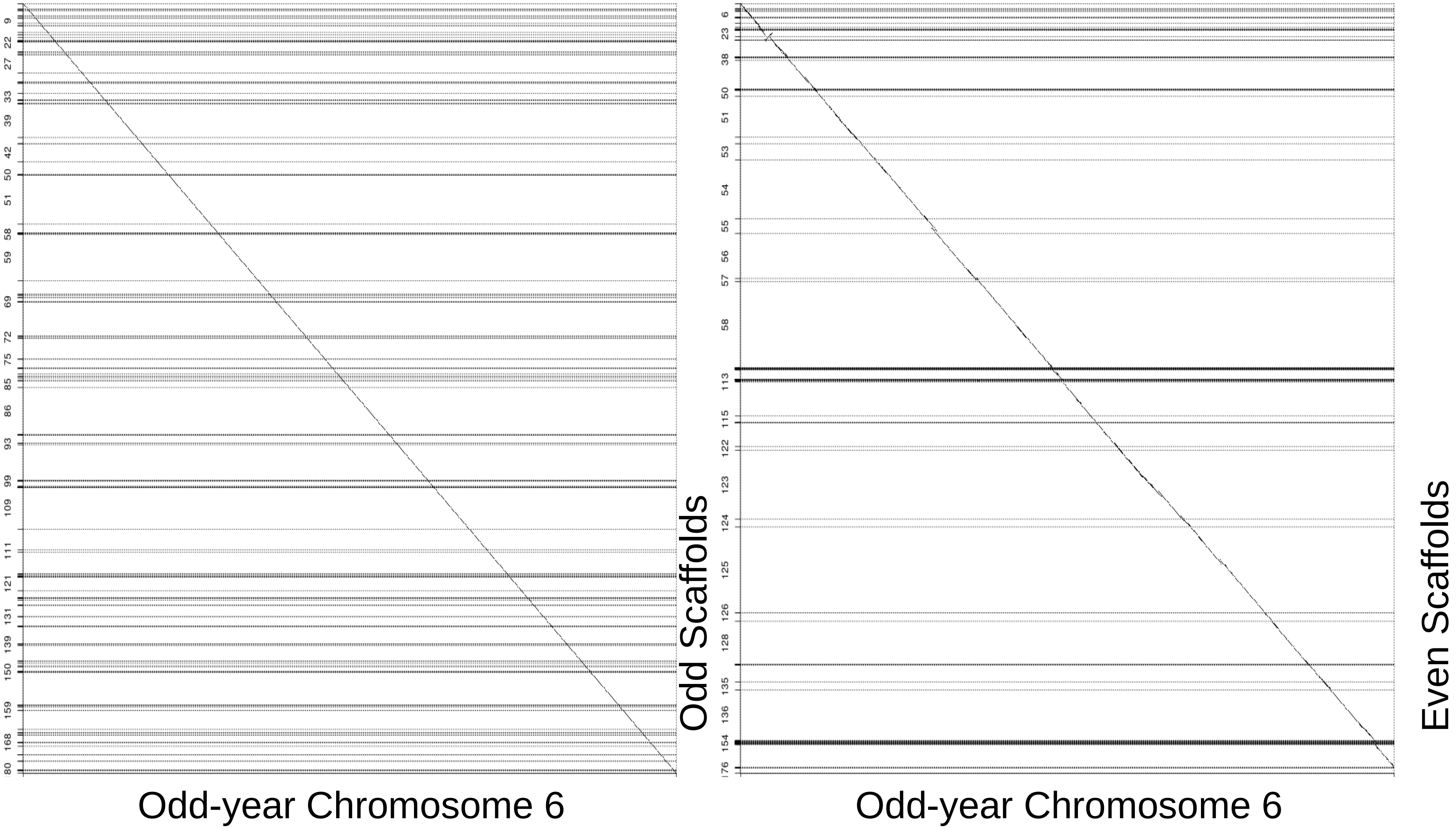

Odd Scaffolds
Odd-year Chromosome 6
Even Scaffolds
Odd-year Chromosome 6

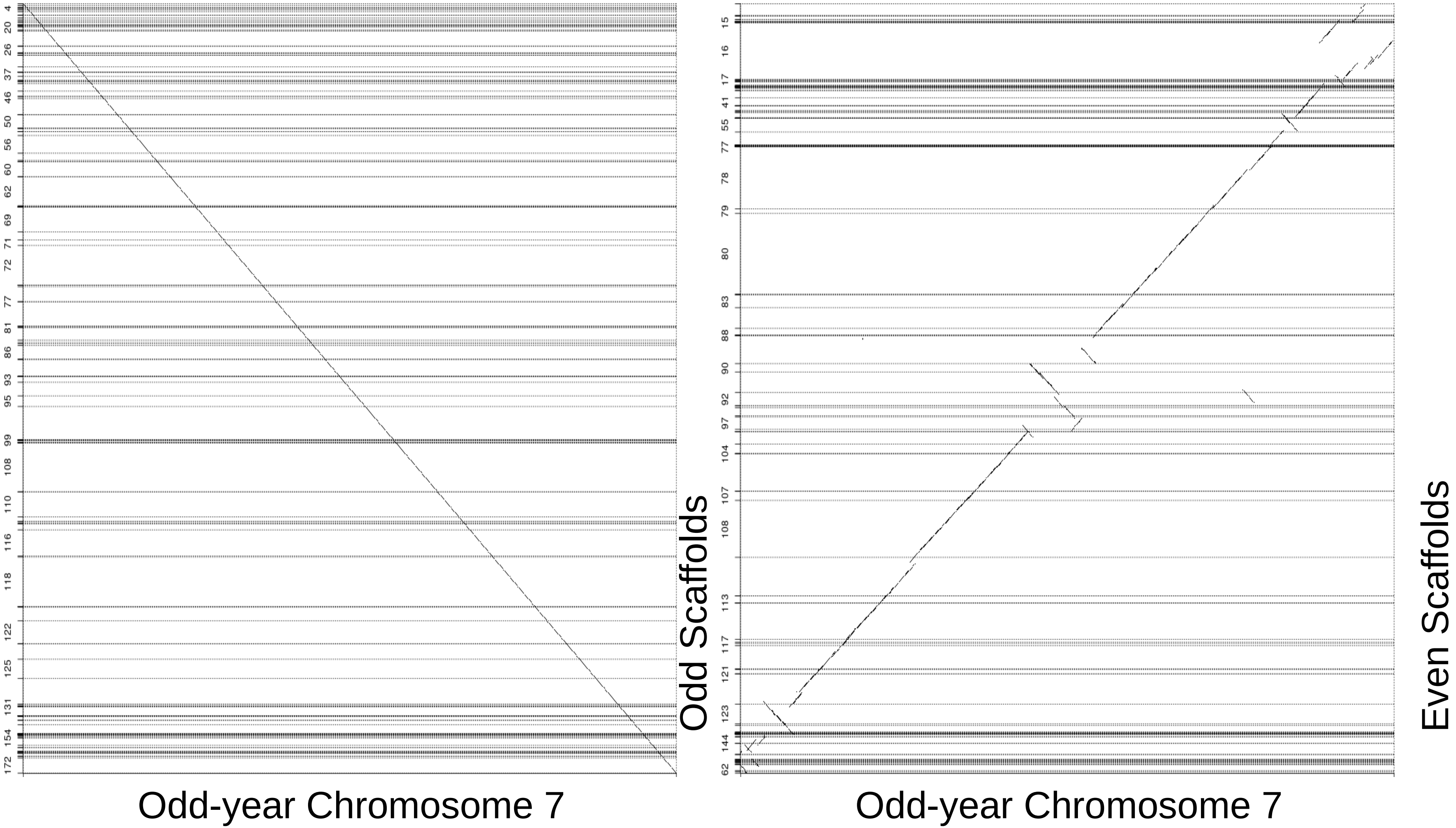

Odd Scaffolds
Odd-year Chromosome 7
4
20
26
37
46
50
56
60
62
69
71
72
77
81
86
93
95
99
108
110
116
118
122
125
131
154
172
Even Scaffolds
Odd-year Chromosome 7
15
16
17
41
55
77
78
79
80
83
88
90
92
97
104
107
108
113
117
121
123
144
62

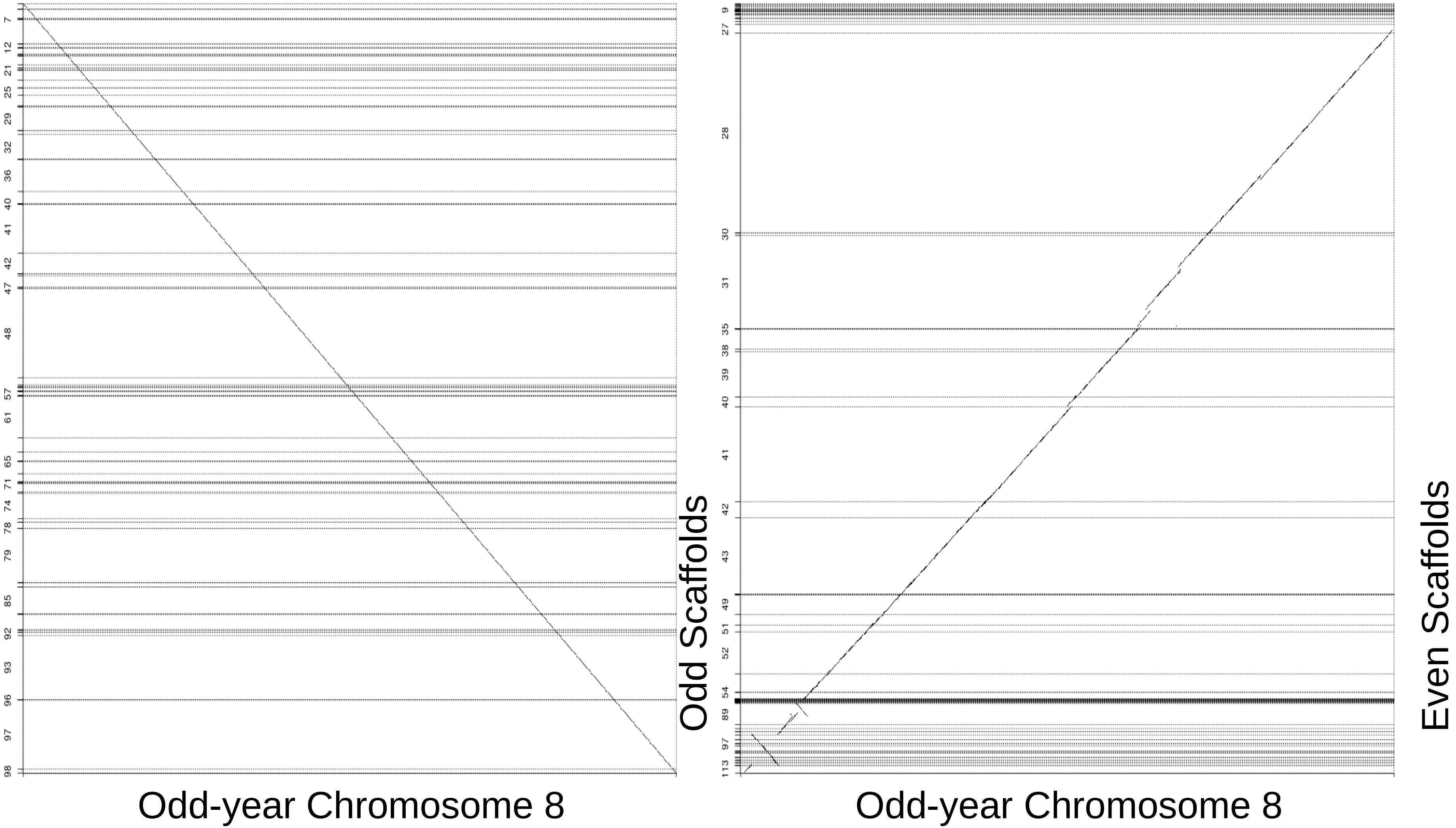
Odd Scaffolds
7
12
21
25
29
32
36
40
41
42
47
48
57
61
65
71
74
78
79
85
92
93
96
97
98
Odd-year Chromosome 8
Even Scaffolds
9
27
28
30
31
35
38
39
40
41
42
43
49
51
52
54
89
97
113
Odd-year Chromosome 8

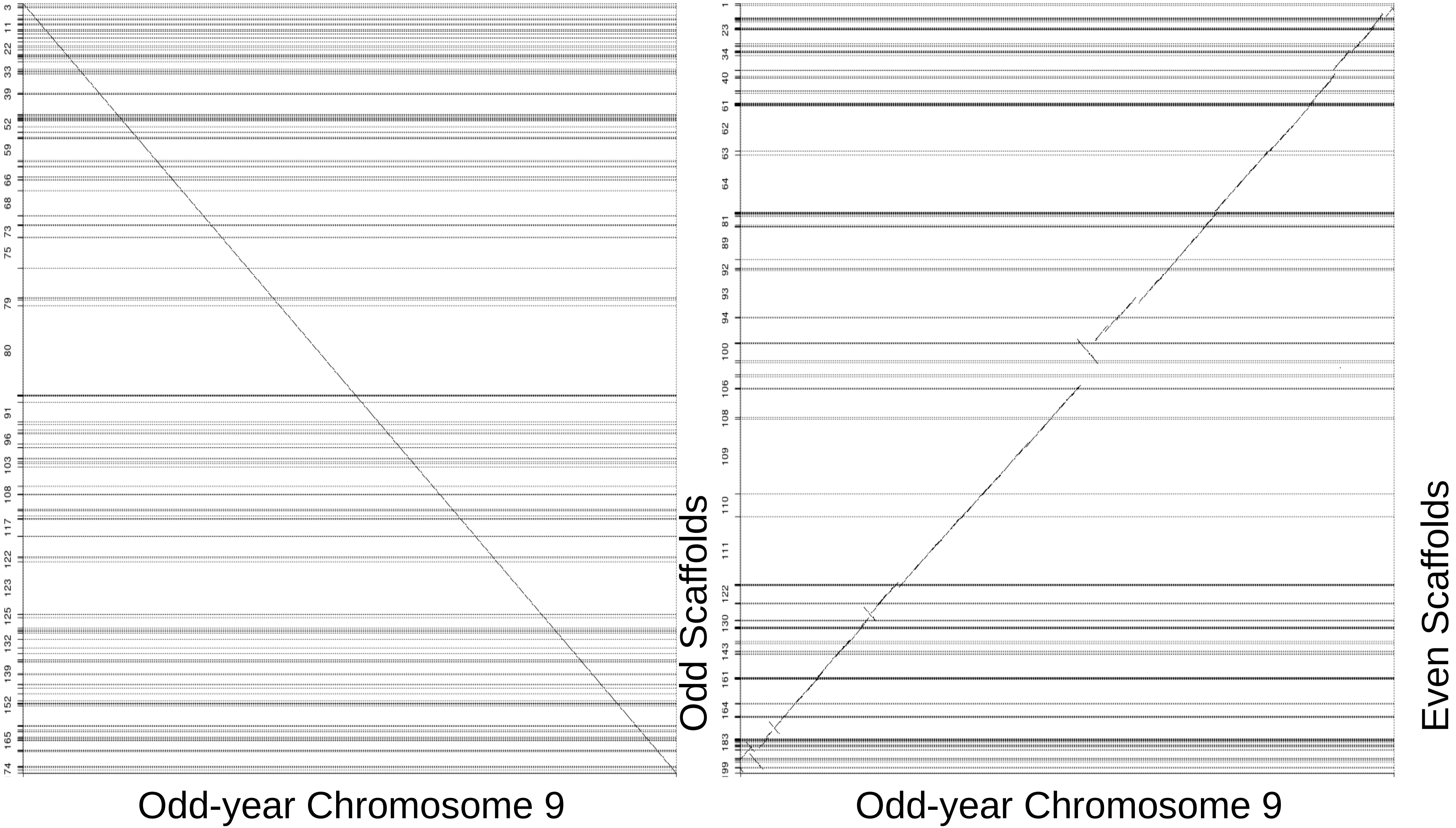

Odd Scaffolds
Odd-year Chromosome 9
Even Scaffolds
Odd-year Chromosome 9

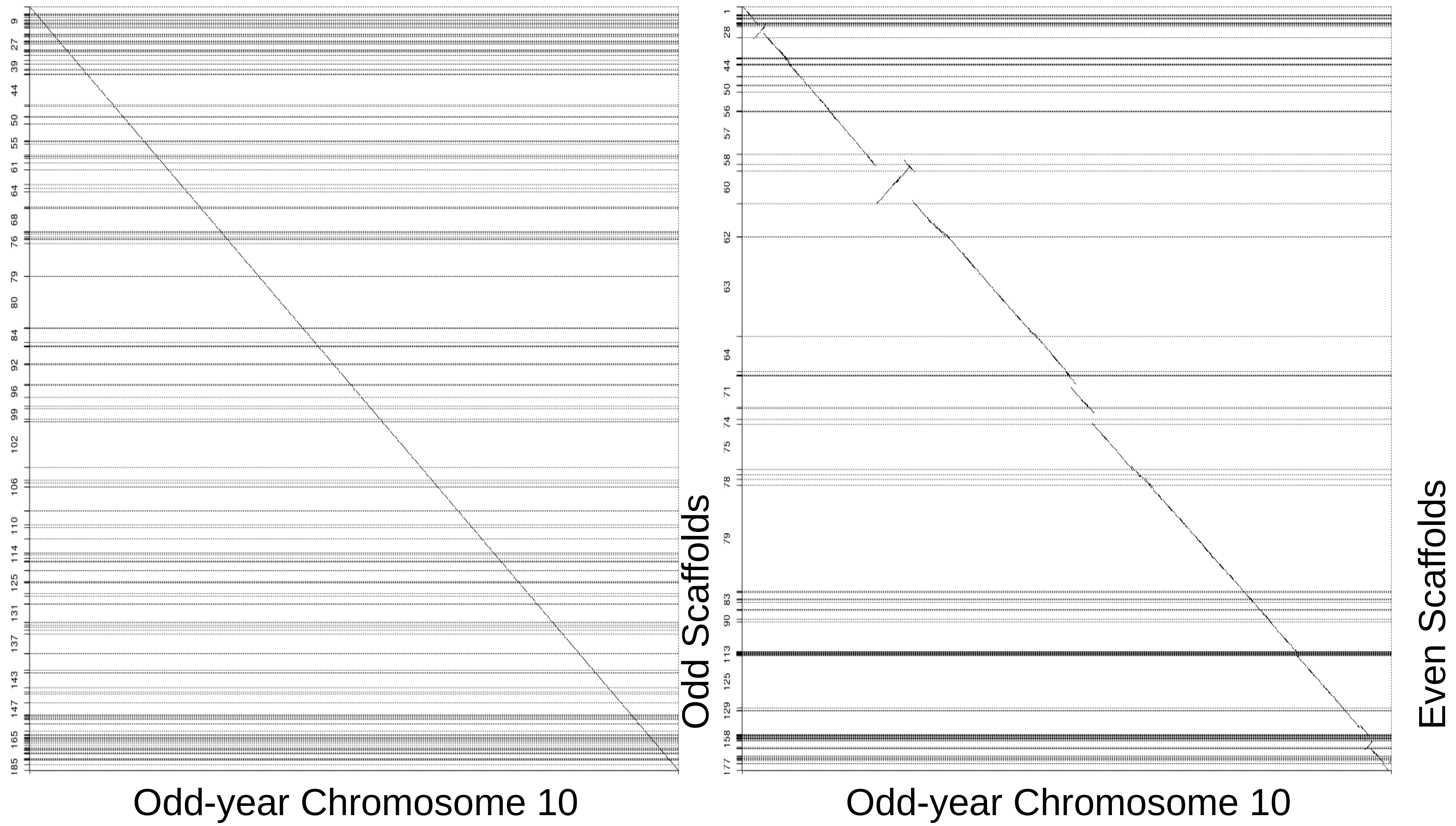
9
27
39
44
50
55
61
64
68
76
79
80
84
92
96
99
102
106
110
114
125
131
137
143
147
165
185
Odd-year Chromosome 10
Odd Scaffolds
1
28
44
50
56
57
58
60
62
63
64
71
74
75
78
79
83
90
113
125
129
158
177
Odd-year Chromosome 10
Even Scaffolds

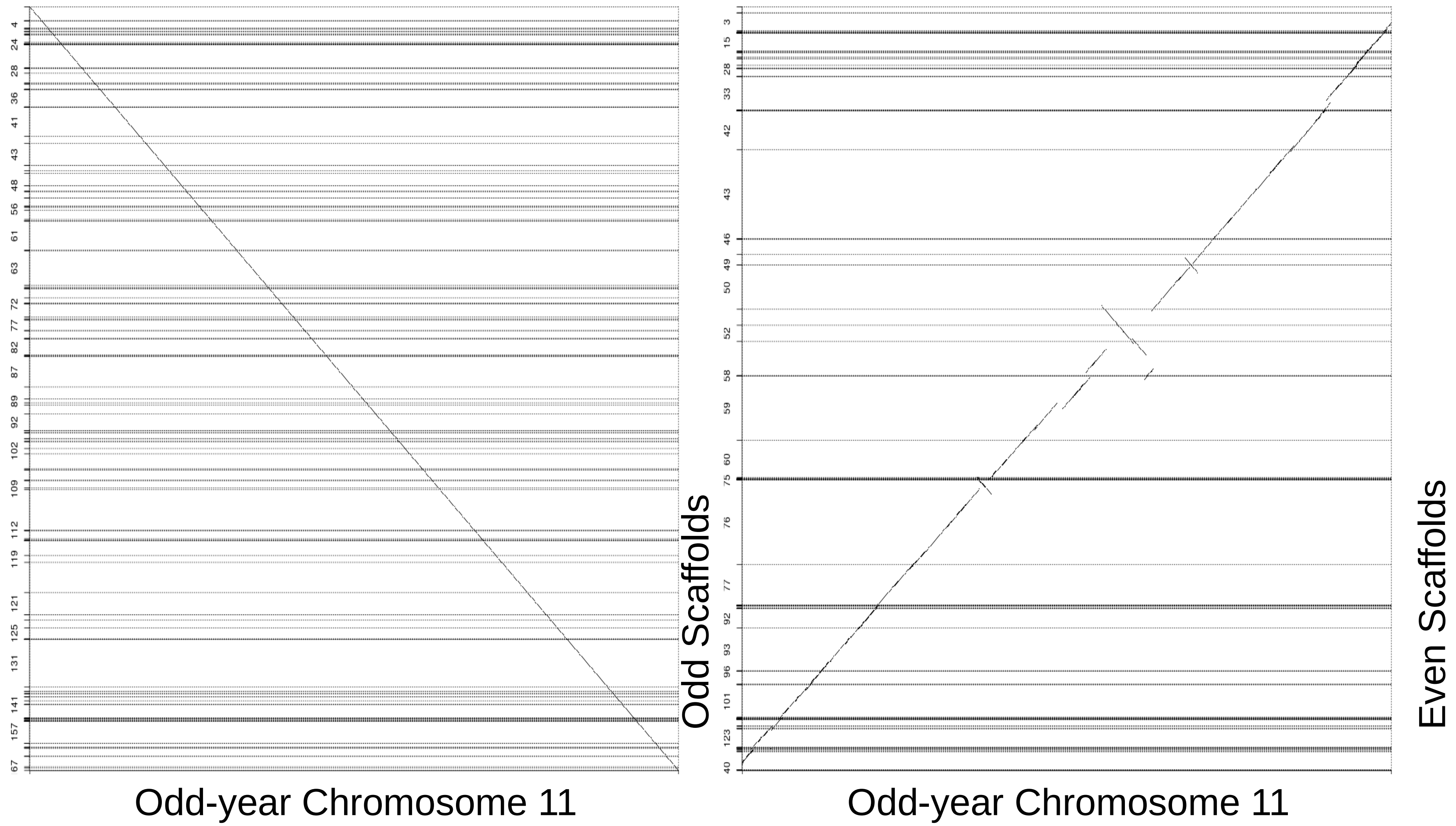
Odd Scaffolds
Odd-year Chromosome 11
Even Scaffolds
Odd-year Chromosome 11

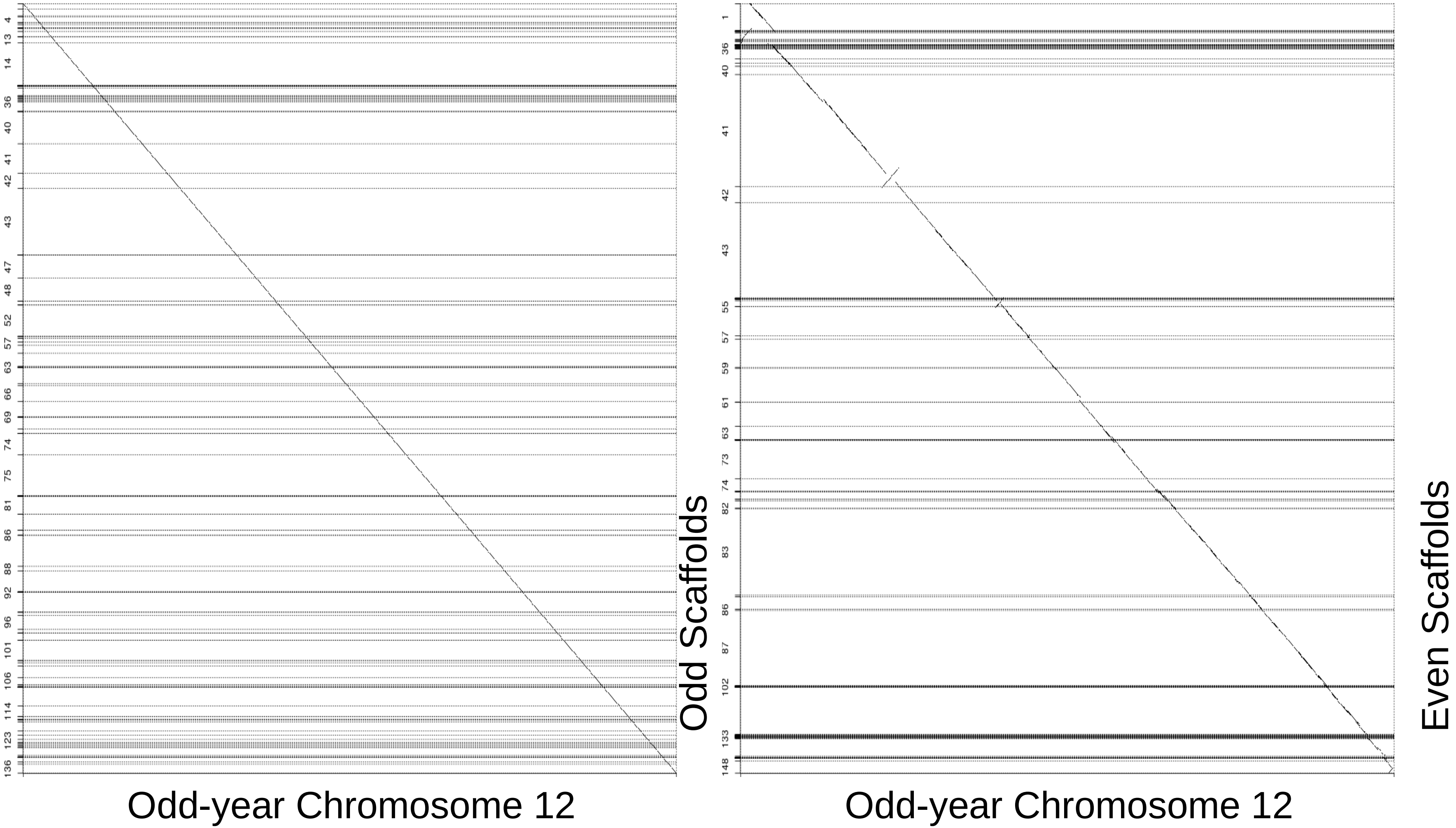
4
13
14
36
40
41
42
43
47
48
52
57
63
66
69
74
75
81
86
88
92
96
101
106
114
123
136
Odd-year Chromosome 12
Odd Scaffolds
1
36
40
41
42
43
55
57
59
61
63
73
74
82
83
86
87
102
133
148
Odd-year Chromosome 12
Even Scaffolds

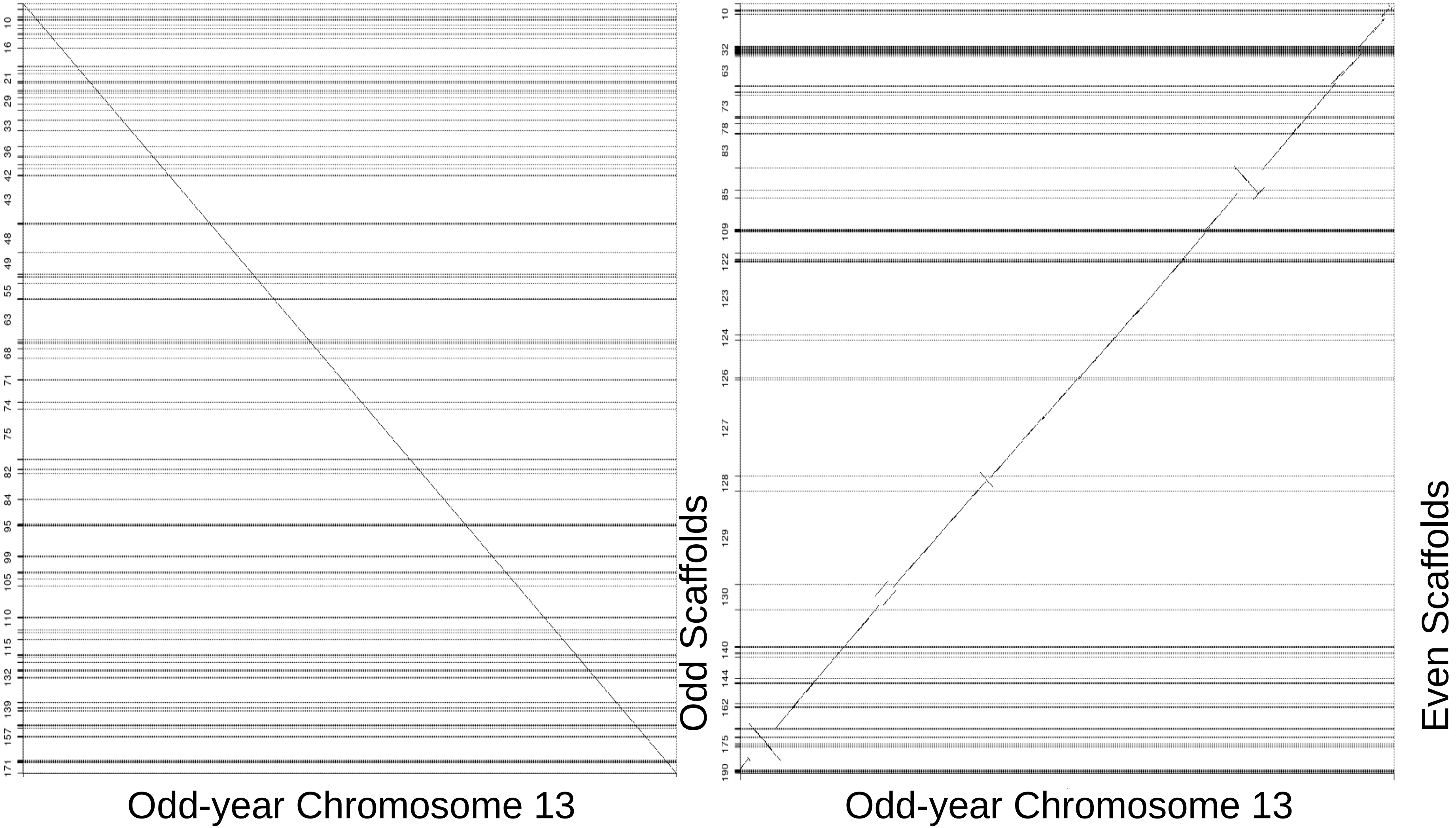

Odd Scaffolds
10
16
21
29
33
36
42
43
48
49
55
63
68
71
74
75
82
84
95
99
105
110
115
132
139
157
171
Odd-year Chromosome 13
Even Scaffolds
10
32
63
73
78
83
85
109
122
123
124
126
127
128
129
130
140
144
162
175
190
Odd-year Chromosome 13

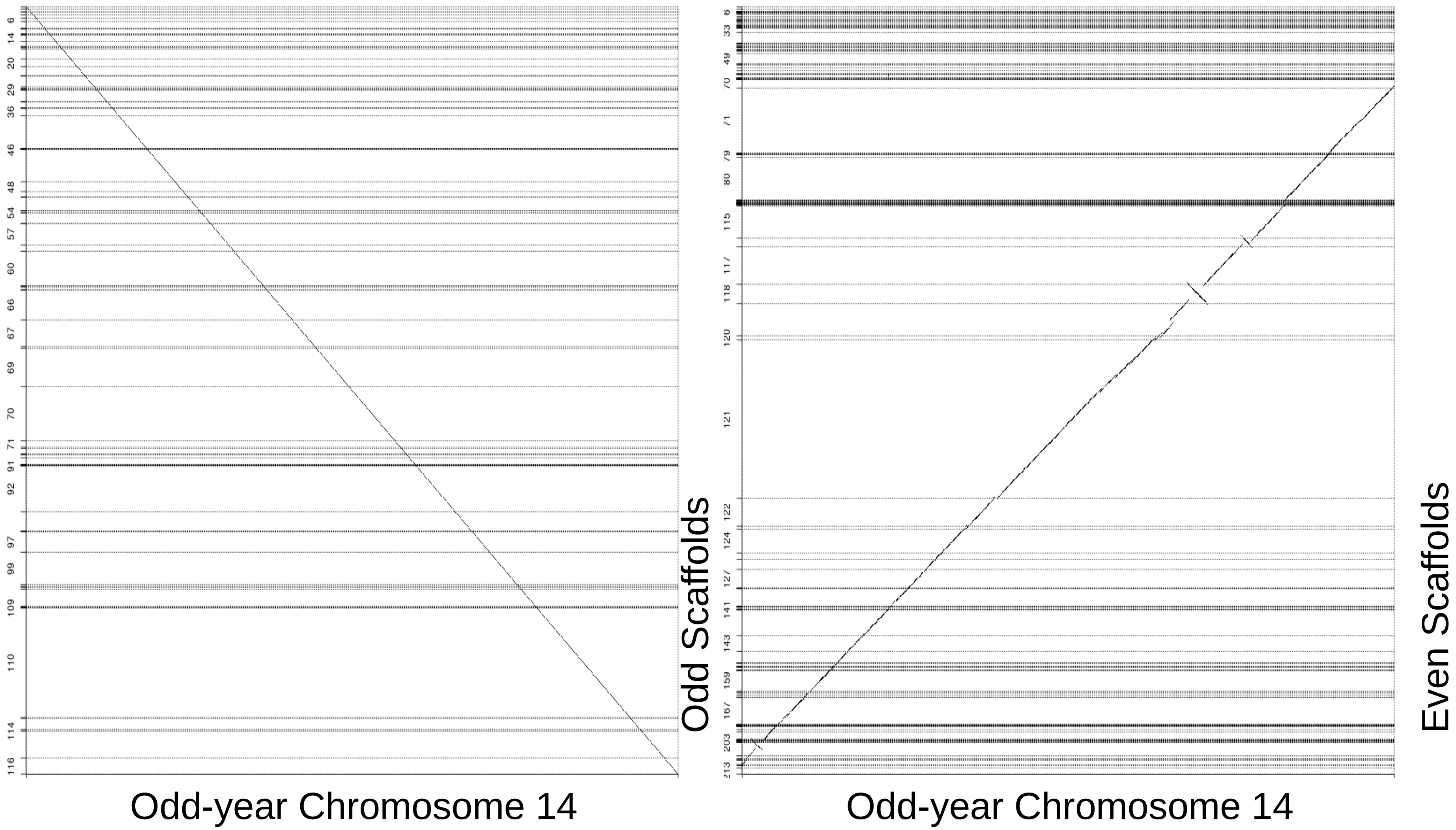
Odd Scaffolds
Odd-year Chromosome 14
Even Scaffolds
Odd-year Chromosome 14

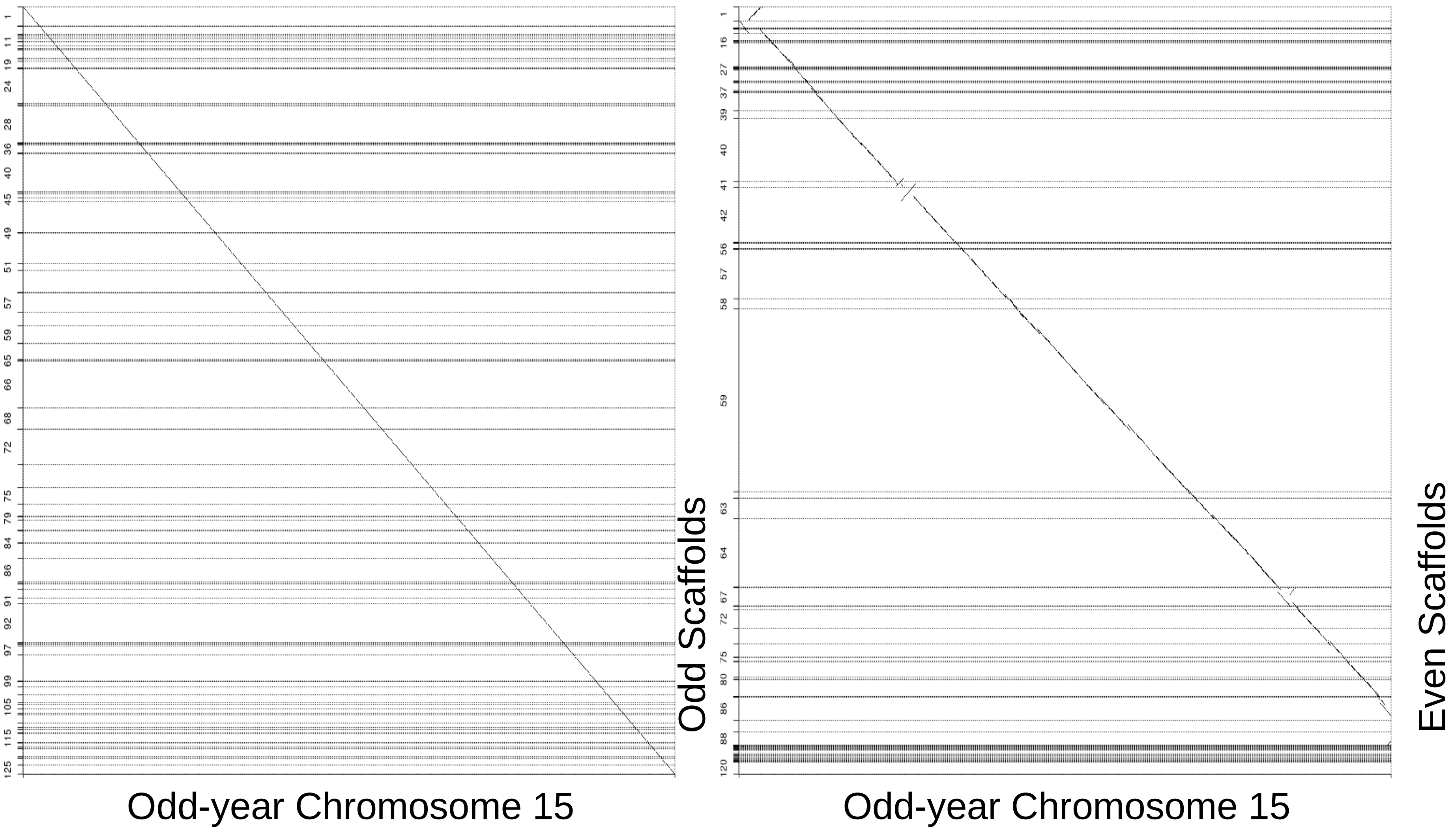
Odd Scaffolds
Odd-year Chromosome 15
Even Scaffolds
Odd-year Chromosome 15

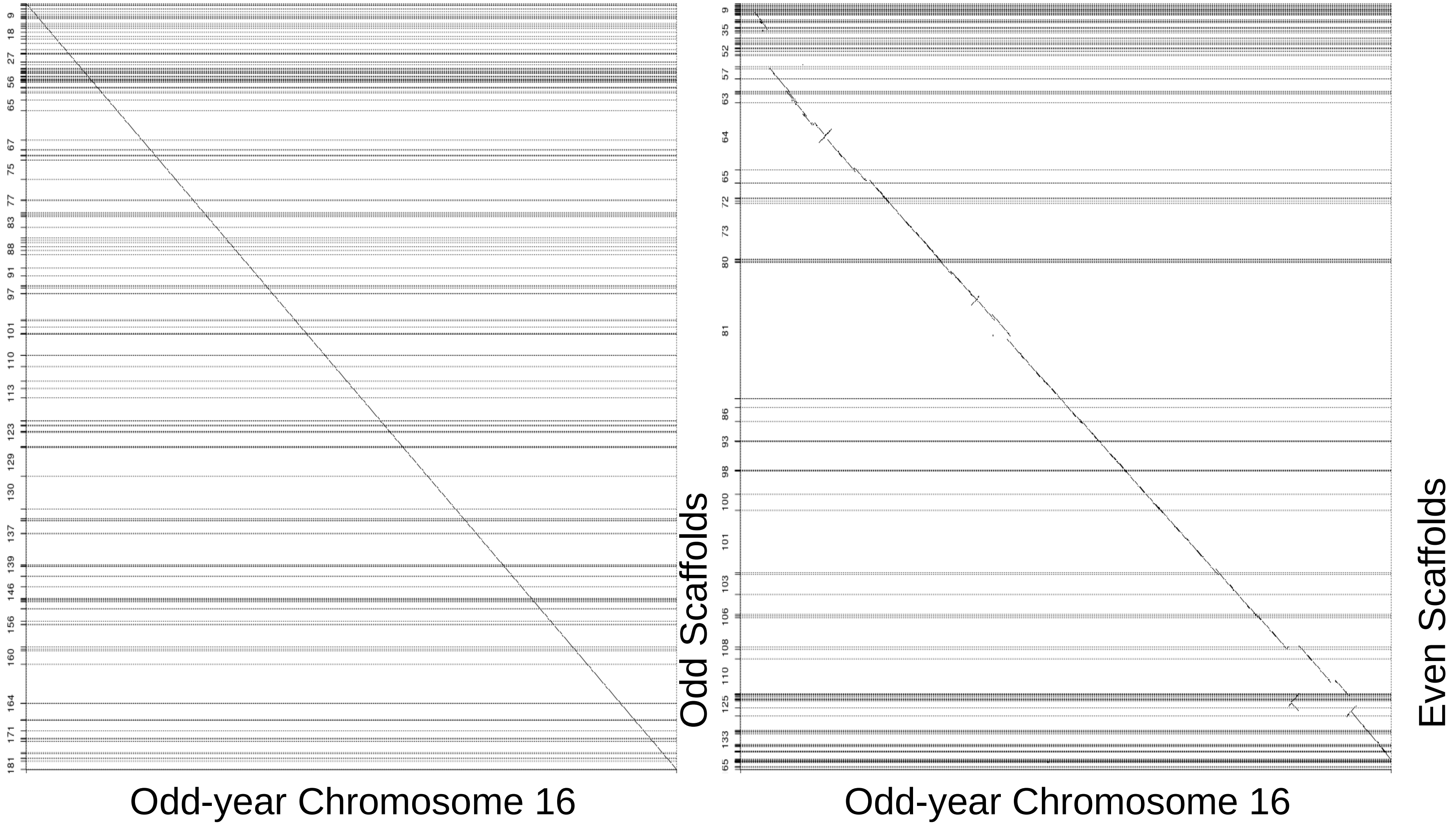
Odd-year Chromosome 16
Odd Scaffolds
9
18
27
56
65
67
75
77
83
88
91
97
101
110
113
123
129
130
137
139
146
156
160
164
171
181
Odd-year Chromosome 16
Even Scaffolds
9
35
52
57
63
64
65
72
73
80
81
86
93
98
100
101
103
106
108
110
125
133
65

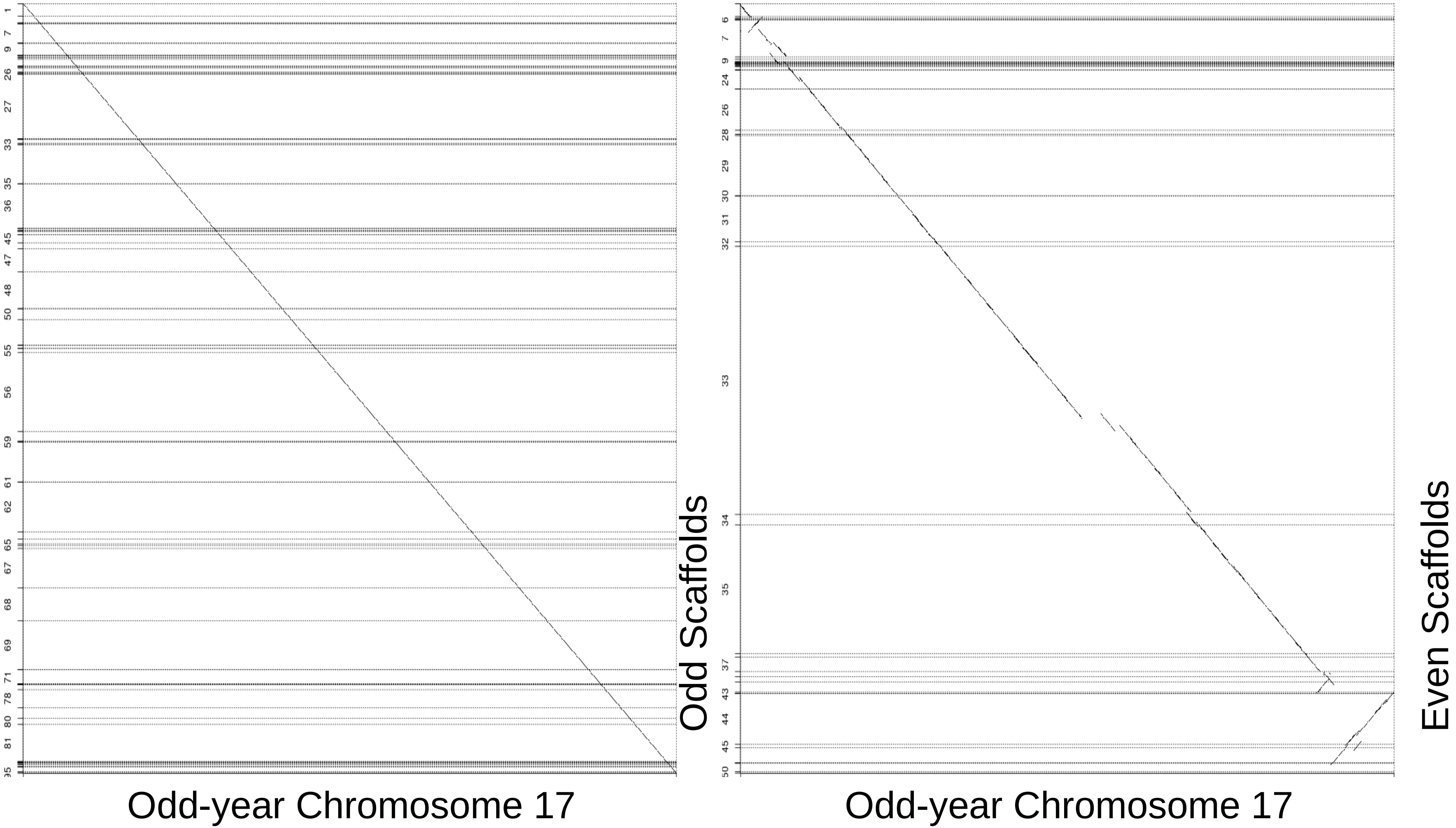
Odd-year Chromosome 17
Odd Scaffolds
Odd-year Chromosome 17
Even Scaffolds

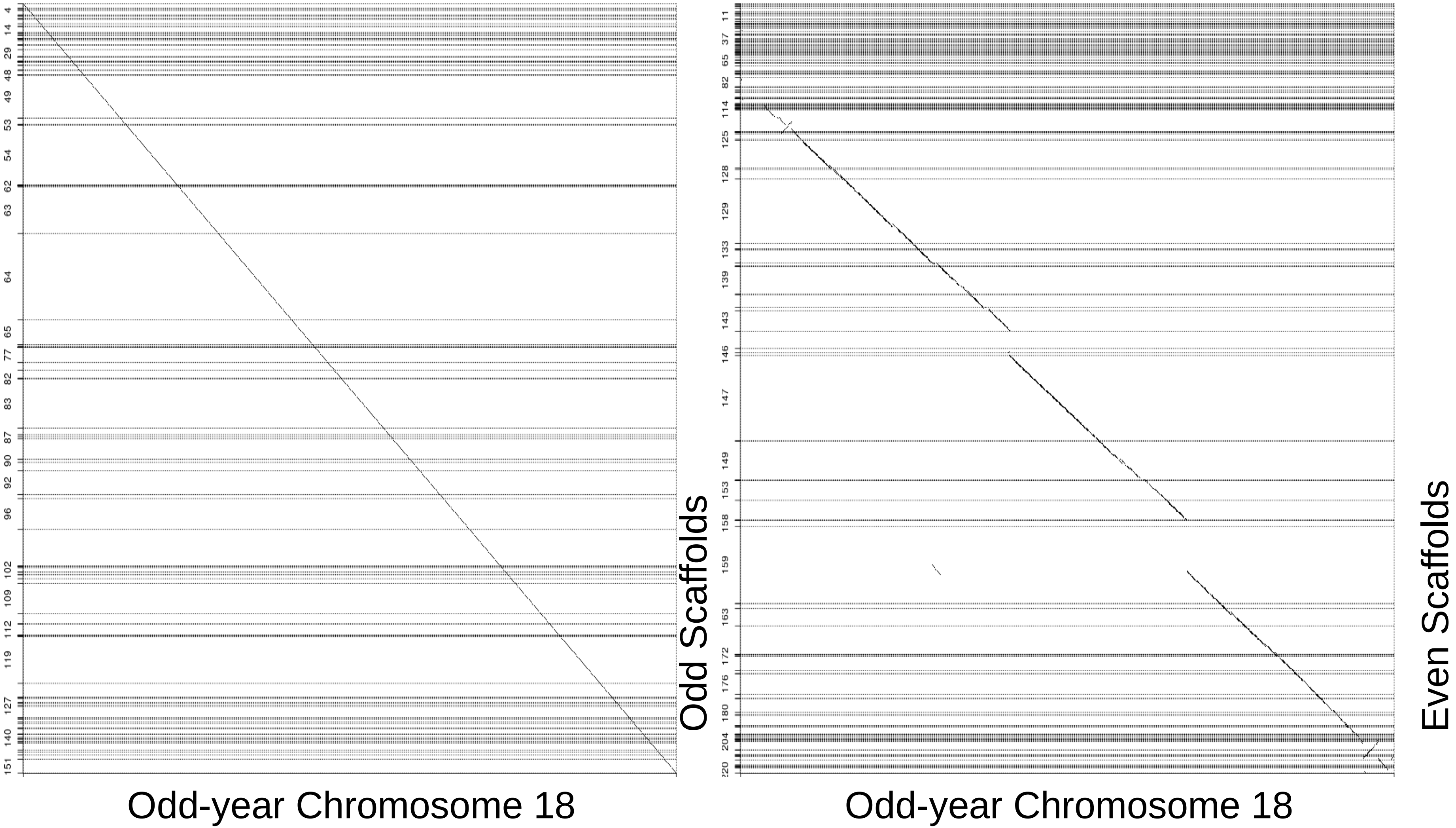

4
14
29
48
49
53
54
62
63
64
65
77
82
83
87
90
92
96
102
109
112
119
127
140
151
Odd-year Chromosome 18
Odd Scaffolds
11
37
65
82
114
125
128
129
133
139
143
146
147
149
153
158
159
163
172
176
180
204
220
Odd-year Chromosome 18
Even Scaffolds

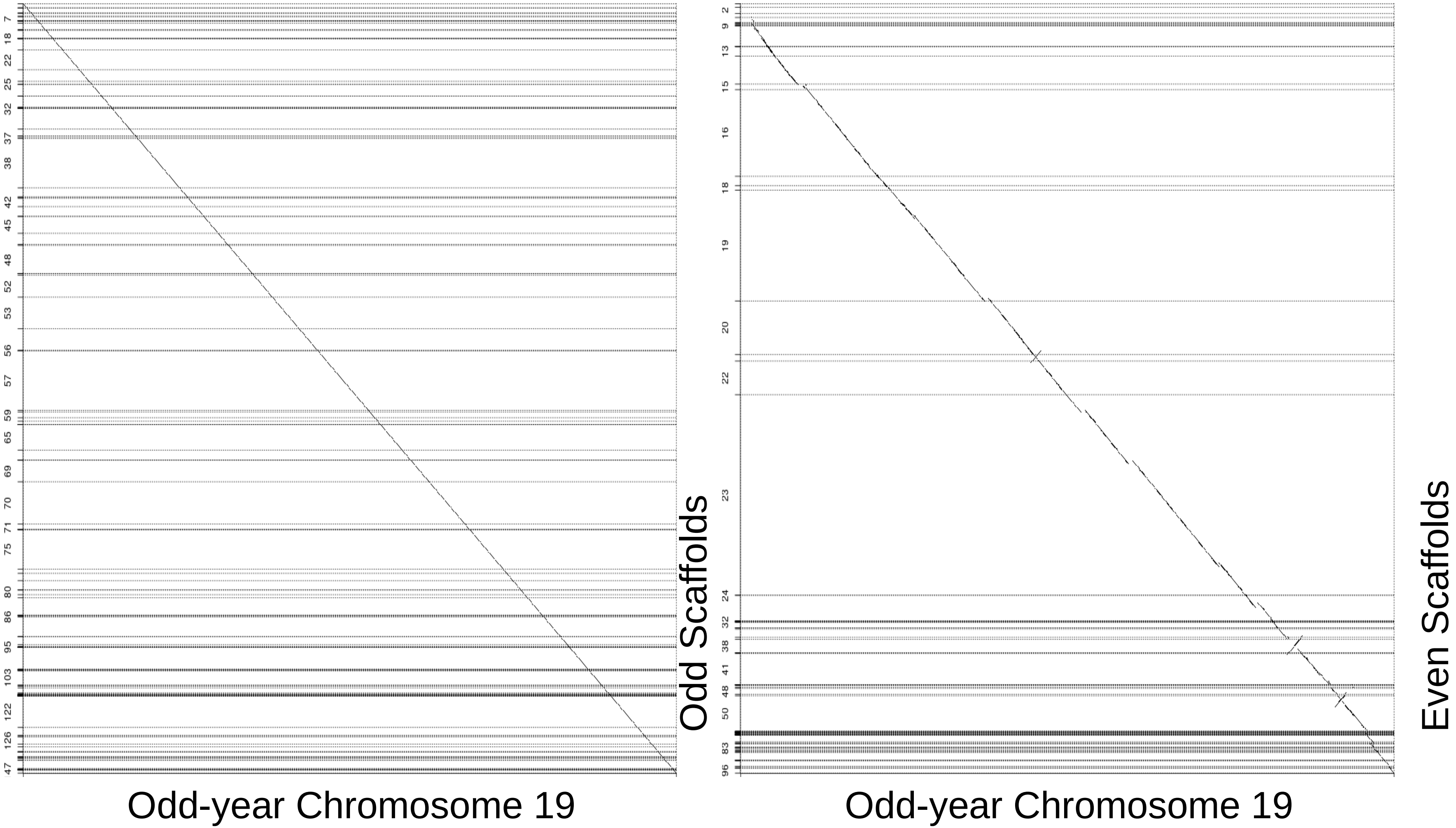
Odd Scaffolds
Odd-year Chromosome 19
Even Scaffolds
Odd-year Chromosome 19
7
18
22
25
32
37
38
42
45
48
52
53
56
57
59
65
69
70
71
75
80
86
95
103
122
126
47
2
9
13
15
16
18
19
20
22
23
24
32
38
41
48
50
83
96

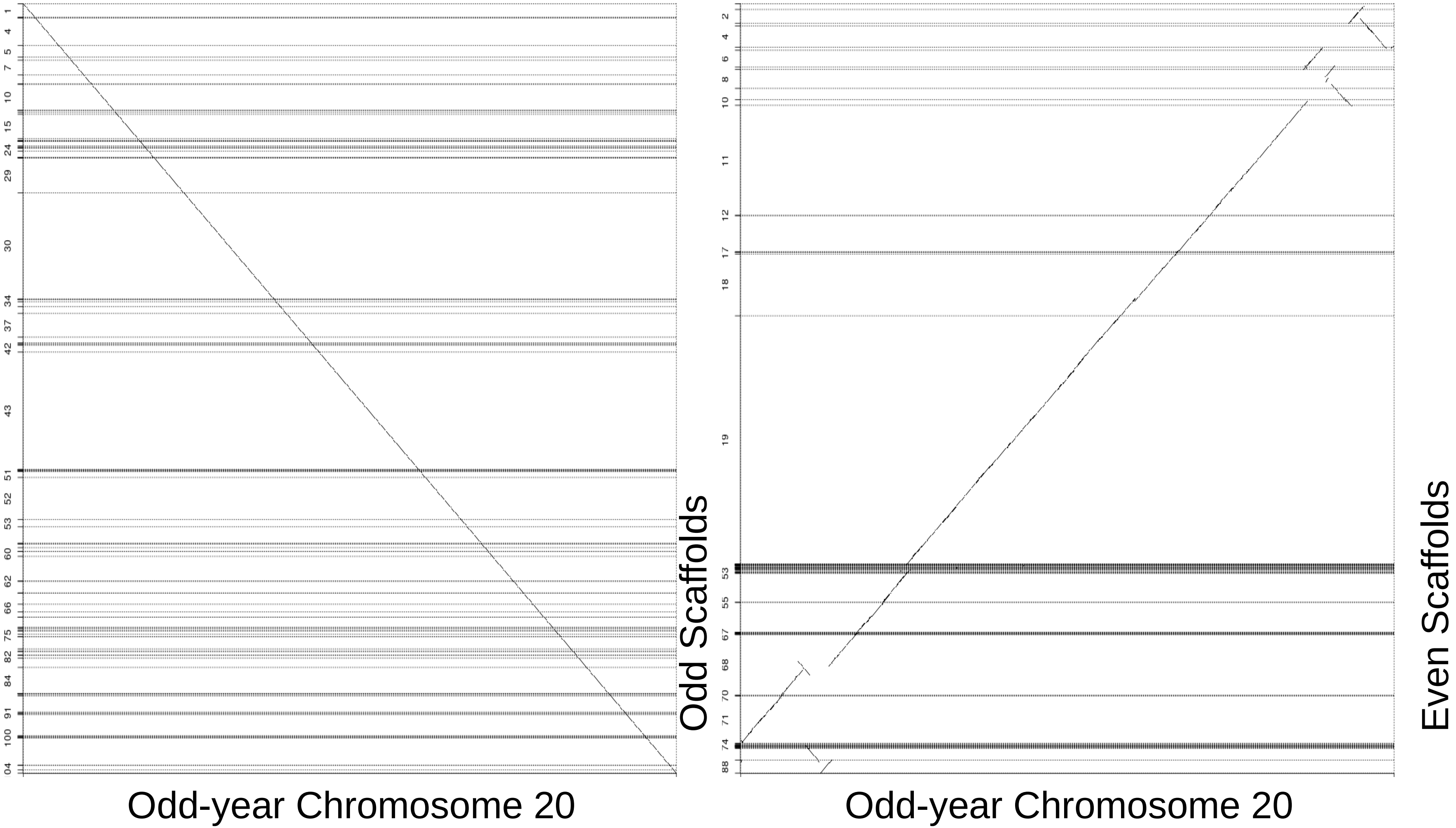
Odd-year Chromosome 20
Odd Scaffolds
Odd-year Chromosome 20
Even Scaffolds

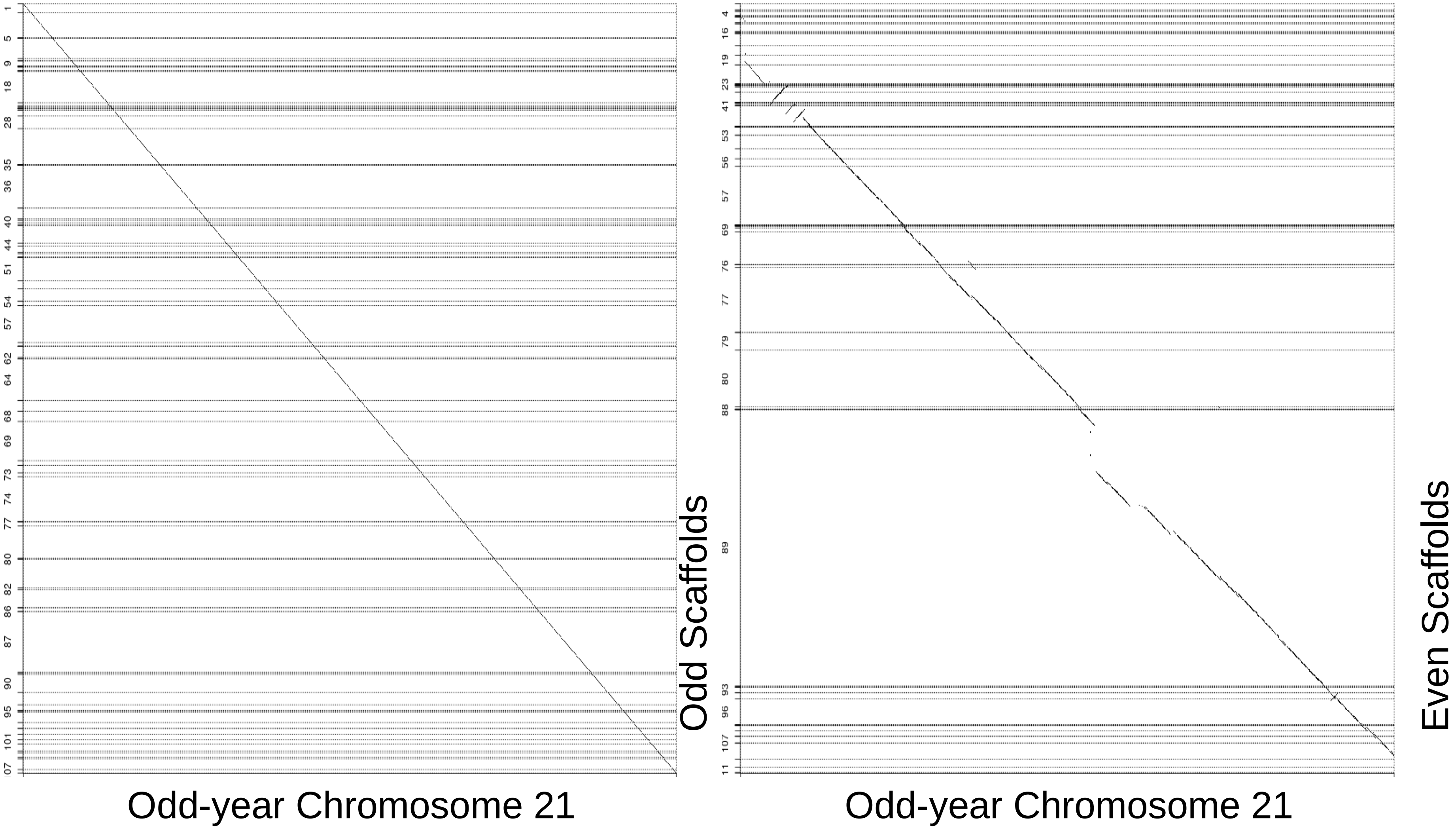
Odd Scaffolds
Odd-year Chromosome 21
Even Scaffolds
Odd-year Chromosome 21

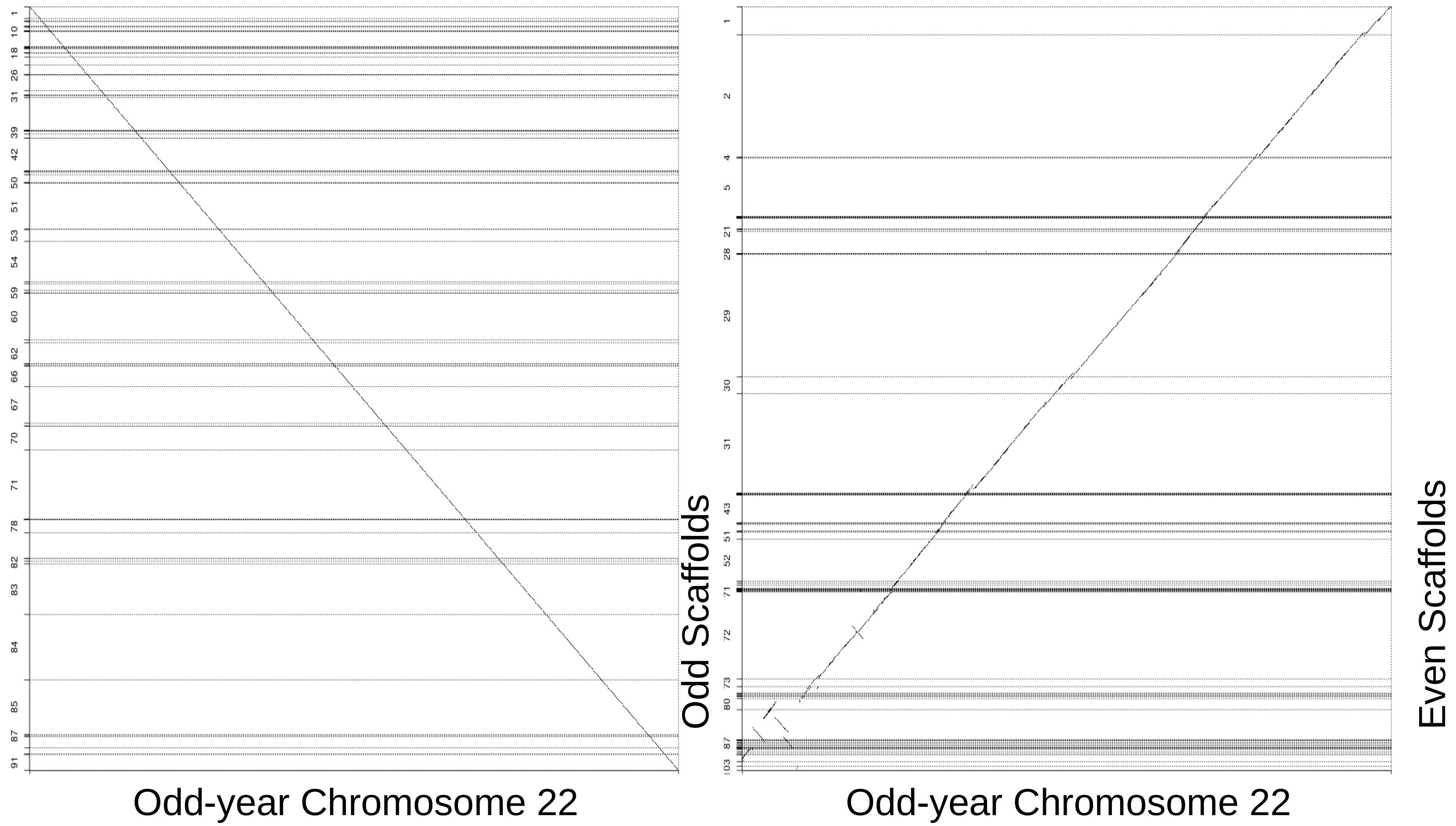

Odd Scaffolds
Even Scaffolds
Odd-year Chromosome 22
Odd-year Chromosome 22
1 10 18 26 31 39 42 50 51 53 54 59 60 62 66 67 70 71 78 82 83 84 85 87 91
1 2 4 5 21 28 29 30 31 43 51 52 71 72 73 80 80 87 103

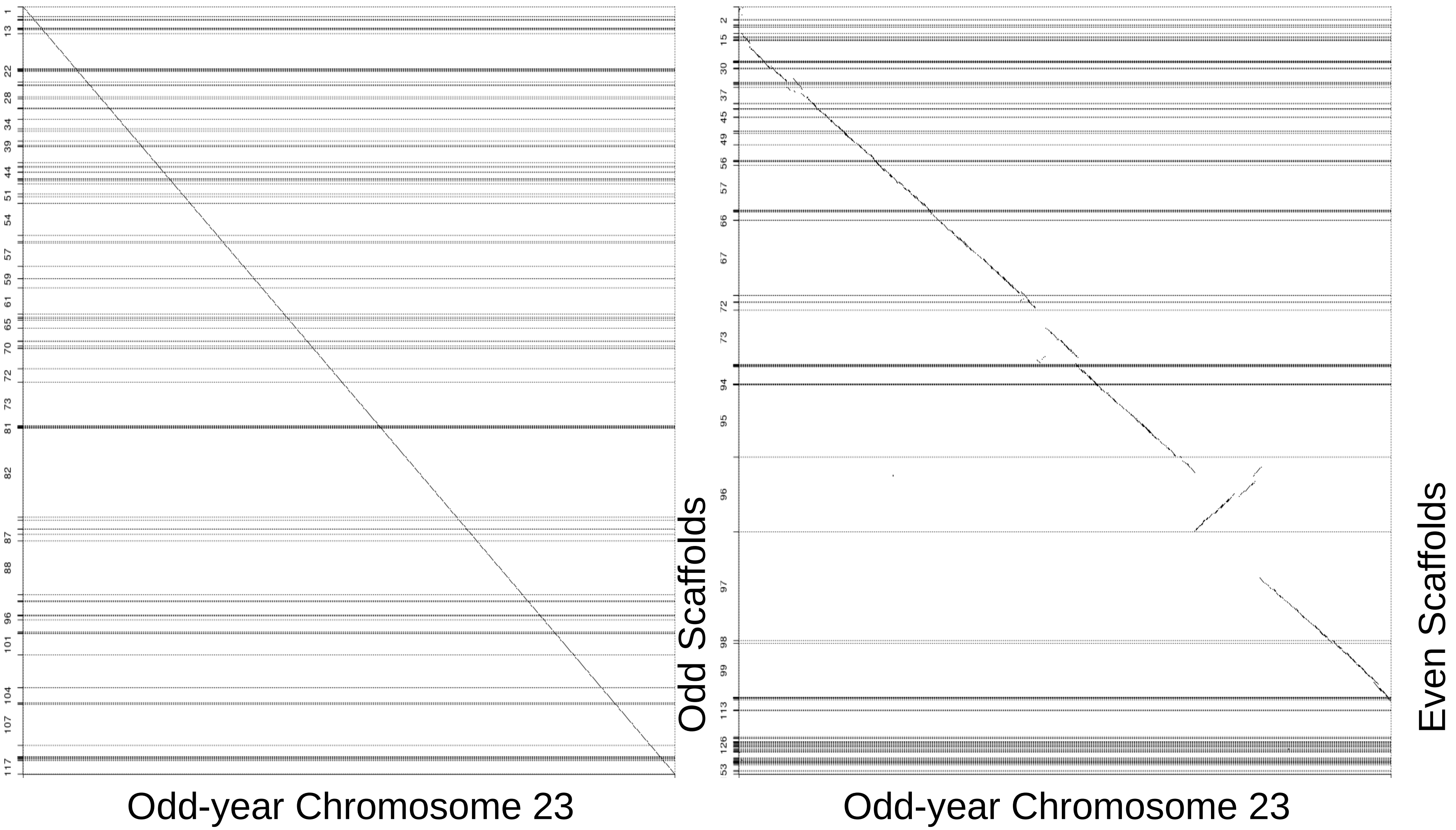
Odd-year Chromosome 23
Odd Scaffolds
Odd-year Chromosome 23
Even Scaffolds

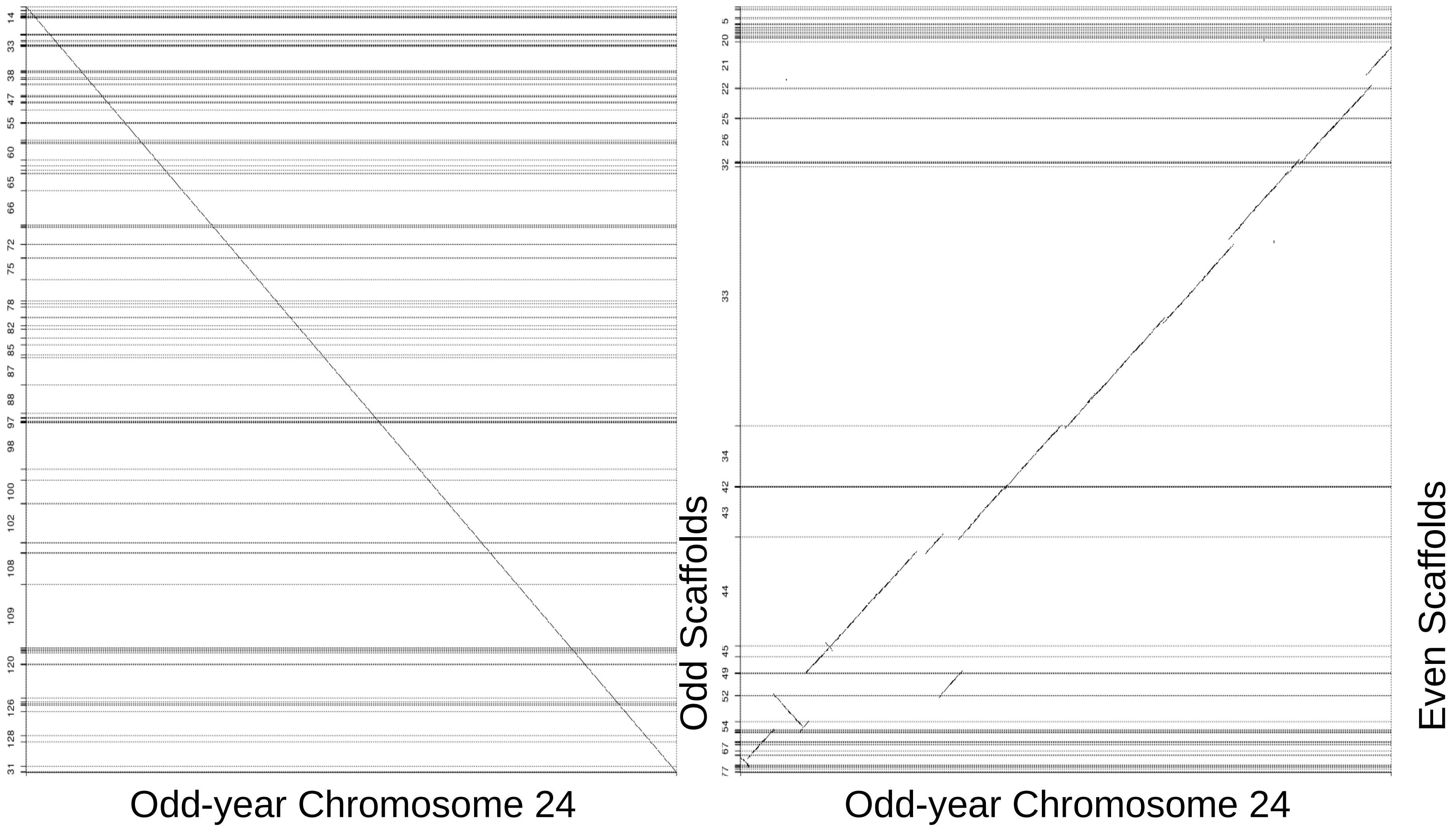
14
33
38
47
55
60
65
66
72
75
78
82
85
87
88
97
98
100
102
108
109
120
126
128
31
Odd-year Chromosome 24
Odd Scaffolds
5
20
21
22
25
26
32
33
34
42
43
44
45
49
52
54
67
77
Odd-year Chromosome 24
Even Scaffolds

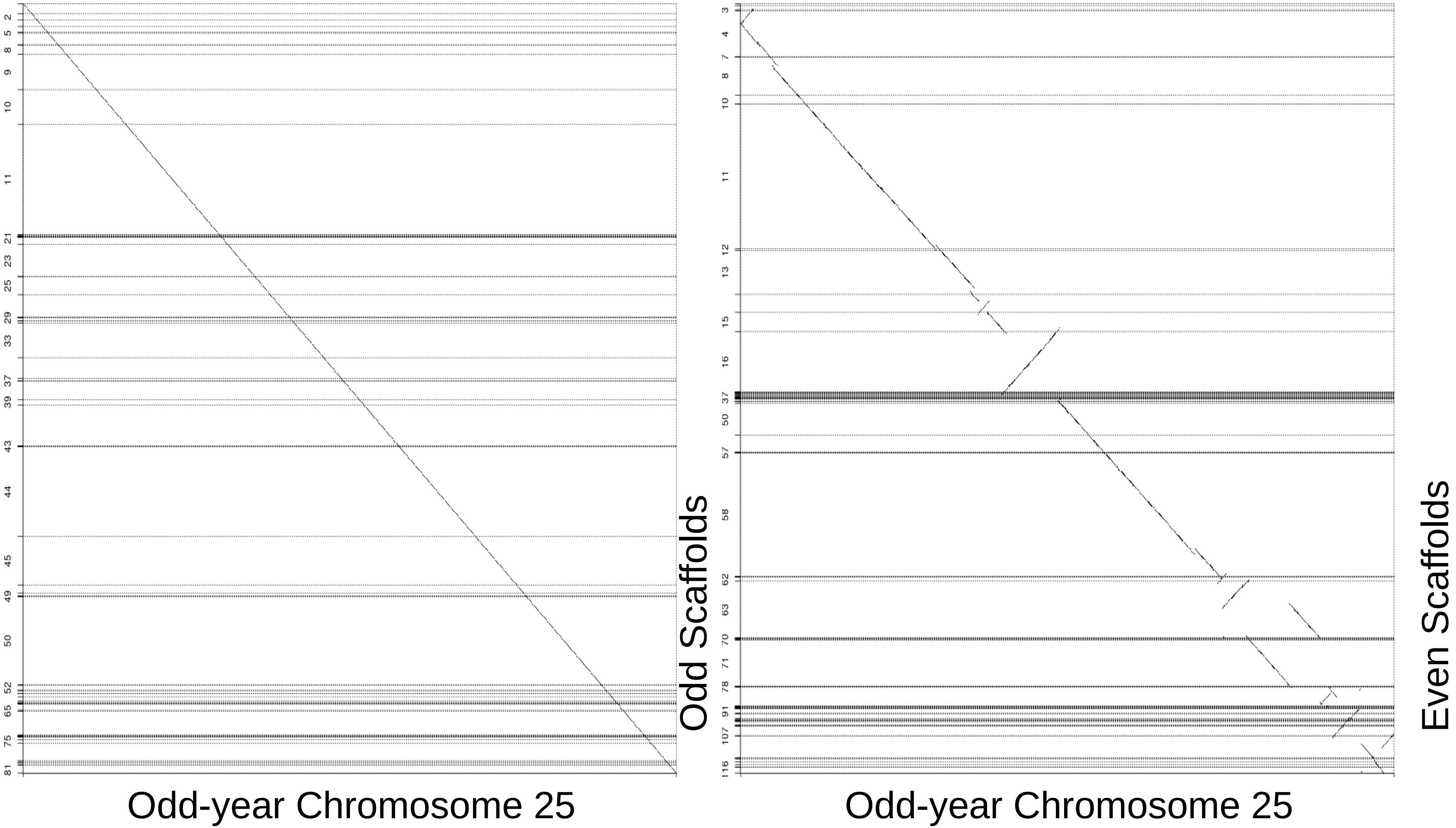
2
5
8
9
10
11
21
23
25
29
33
37
39
43
44
45
49
50
52
65
75
81
Odd-year Chromosome 25
Odd Scaffolds
3
4
7
8
10
11
12
13
15
16
37
50
57
58
62
63
70
71
78
91
107
116
Odd-year Chromosome 25
Even Scaffolds

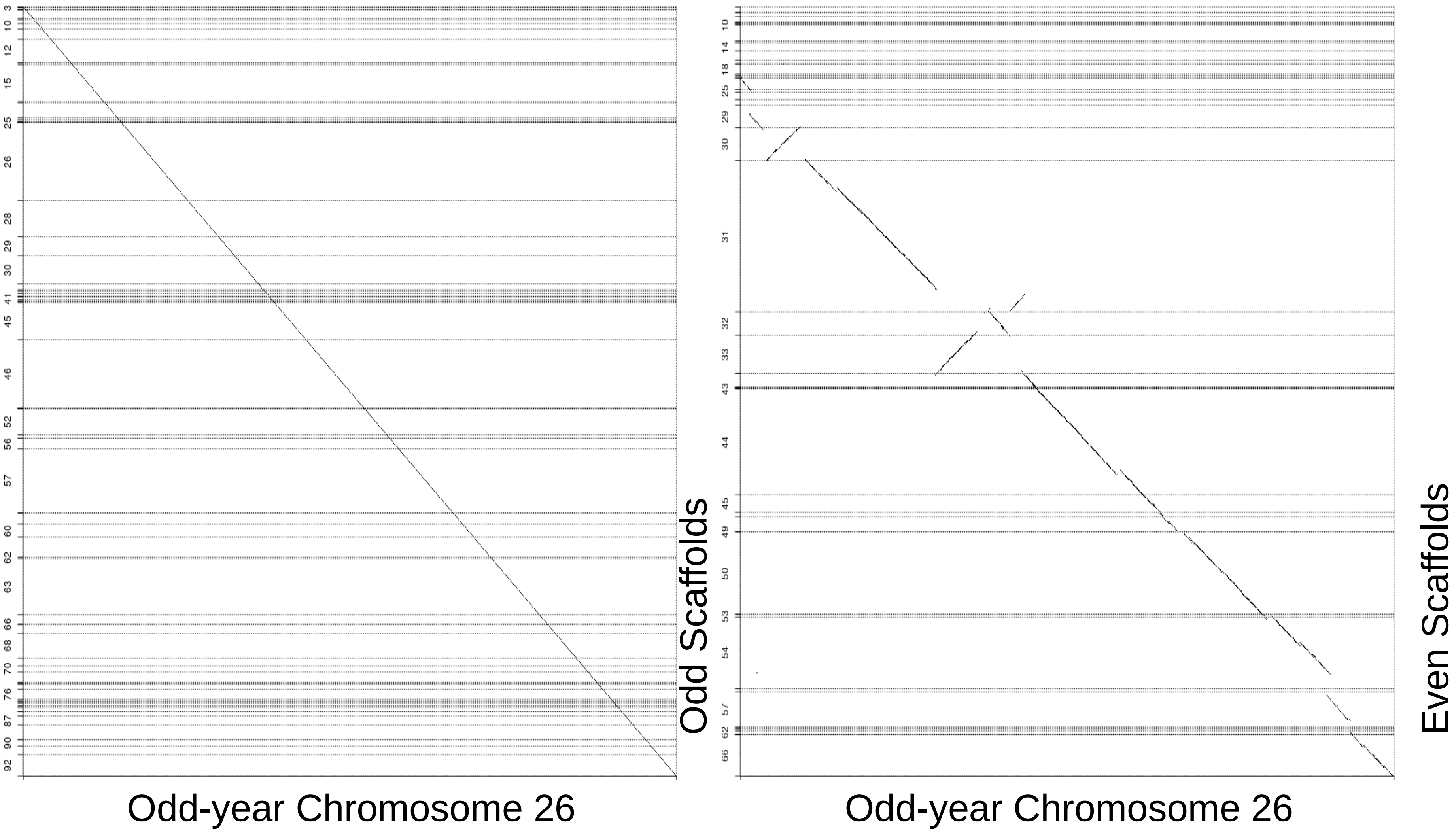
3
10
12
15
25
26
28
29
30
41
45
46
52
56
57
60
62
63
66
68
70
76
87
90
92
Odd-year Chromosome 26
Odd Scaffolds
10
14
18
25
29
30
31
32
33
43
44
45
49
50
53
54
57
62
66
Odd-year Chromosome 26
Even Scaffolds
