# Supplementary figures and images for: The pink salmon genome: Uncovering the genomic consequences of a two-year life cycle
Source: PLoS One. 2021 Dec 17;16(12):e0255752. doi: 10.1371/journal.pone.0255752 (PMC8682878; doi:10.1371/journal.pone.0255752)

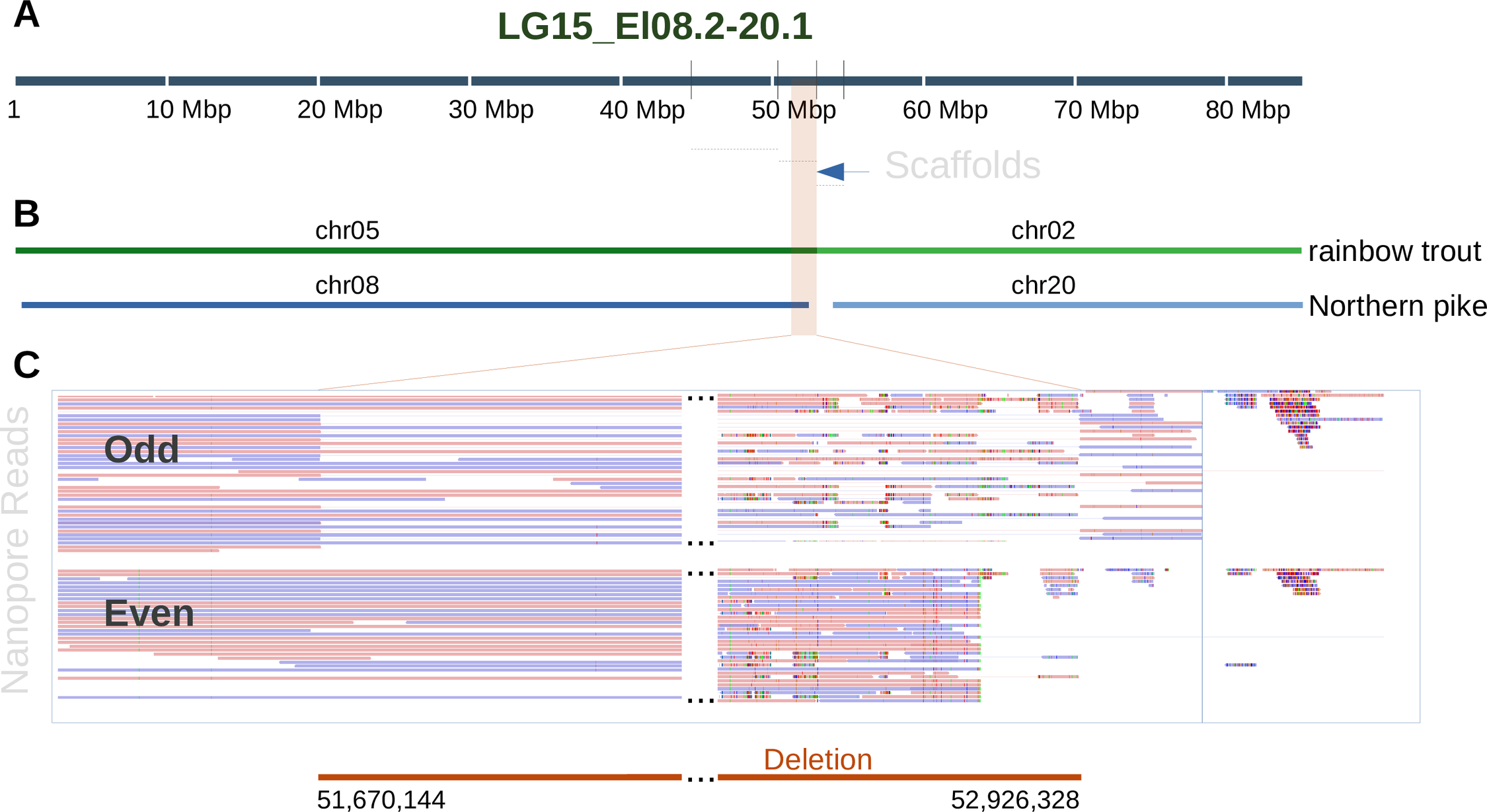

Supplement: S2 Fig — Depiction of LG15_El08.2–20.1 and a chromosomal polymorphism, either a deletion or evidence of a chromosomal fusion. A) LG15_El08.2–20.1 is depicted with the distance and location of the purposed polymorphism (in light translucent red). Scaffolds/contigs that comprise the region surrounding the polymorphism are shown below the chromosomal depiction, with a blue arrow showing where multiple small contigs were placed. B) Synteny with rainbow trout and Northern pike is shown based on CHROMEISTER [111] alignments. C) ONT/Nanopore reads that were used to generate the genome assemblies were aligned back to the odd-year genome and visualized with IGV. Reads in the odd-year individual are shown flanking the deletion (the display was split because the region was too large to adequately visualize continuously, ellipses mark the split). The proposed deletion is shown below the long reads. (TIF) [file pone.0255752.s003.tif]

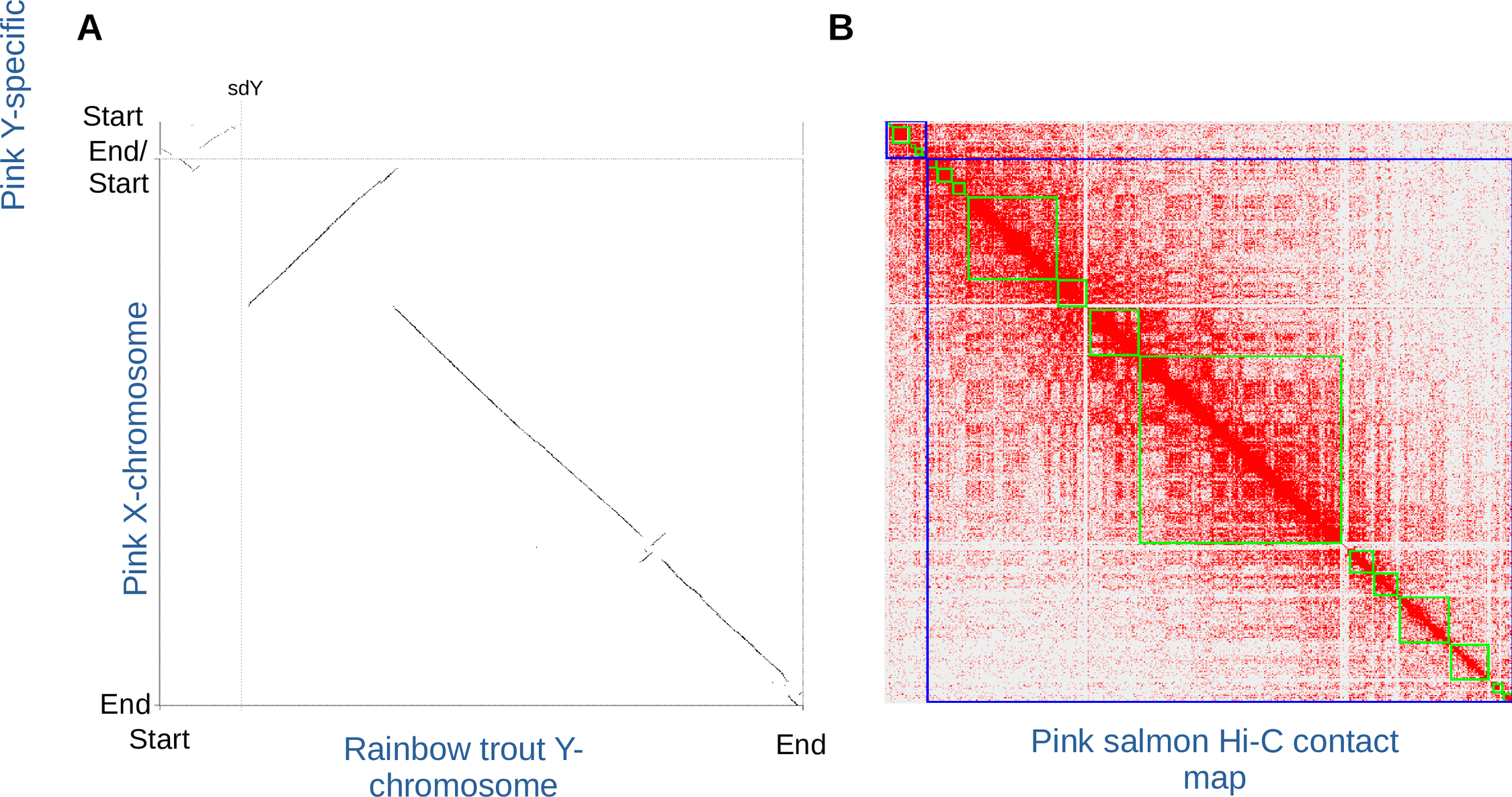

Supplement: S3 Fig — A) A CHROMEISTER [111] dotplot between the Y-specific portion (top) and shared portion (bottom) of LG20_El14.2 of the even-year pink salmon genome assembly and the rainbow trout Y-chromosome [65]. The location of the sdY gene is shown based on the position in the rainbow trout chromosome. B) A plot of the Hi-C contact map of the even-year pink salmon genome assembly produced by Juicebox [66]. The blue boxes represent chromosomes/pseudomolecules (the top is the proposed Y-specific region and the bottom is the rest of LG20_El14.2) and the green boxes represent scaffolds or contigs mapped to this chromosome. Red points represent contacts (close proximity) between regions. There are multiple inversions between the pink salmon and rainbow trout genome seen in the dotplot, but the contact map supports the order and orientation for the pink salmon genome assembly and these could represent actual inversions between species instead of assembly errors. (TIF) [file pone.0255752.s004.tif]

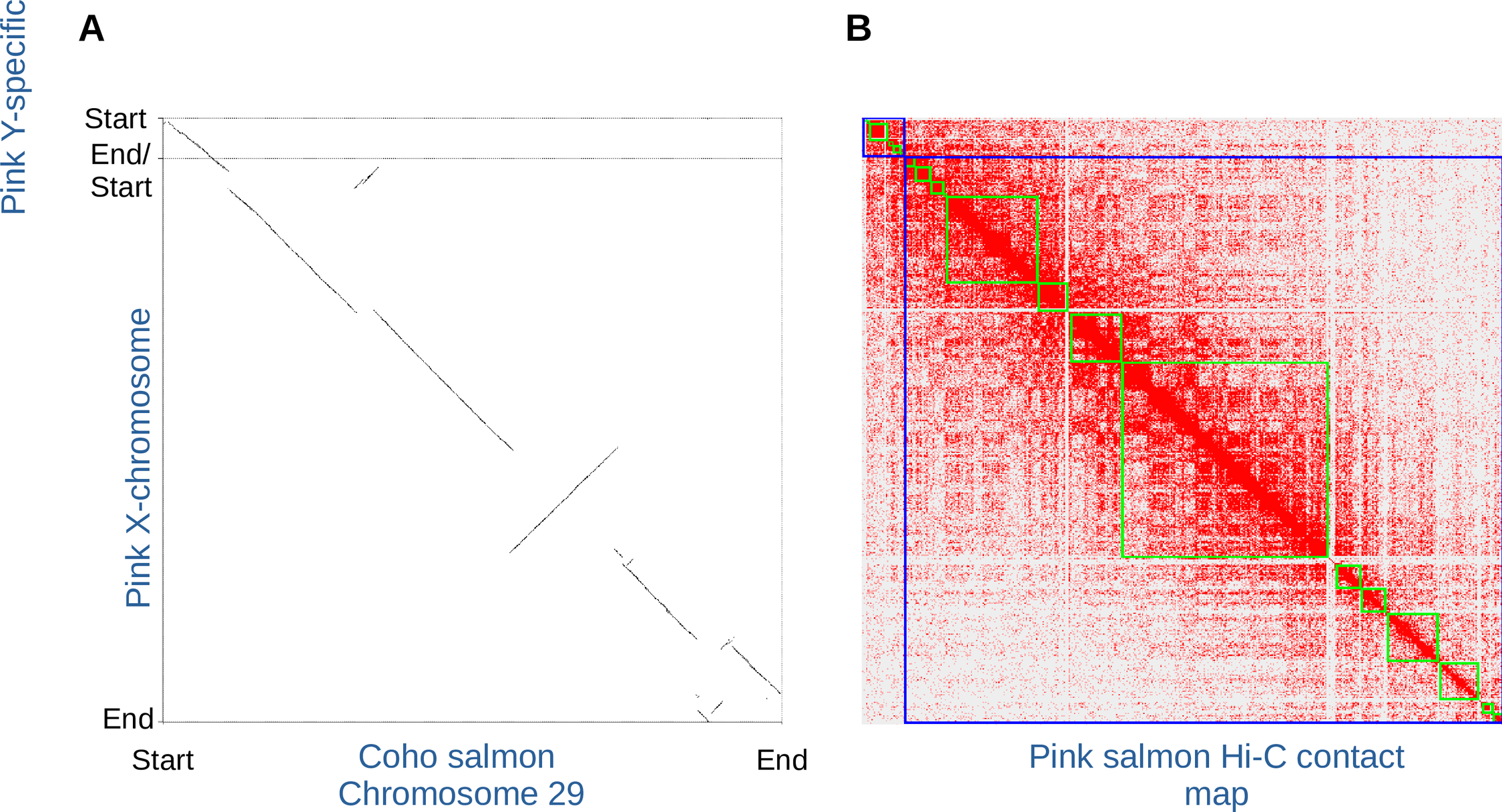

Supplement: S4 Fig — A) A CHROMEISTER [111] dotplot between the Y-specific portion (top) and shared portion (bottom) of LG20_El14.2 of the even-year pink salmon genome assembly and coho salmon chromosome 29. B) A plot of the Hi-C contact map of the even-year pink salmon genome assembly produced by Juicebox [66]. The blue boxes represent chromosomes/pseudomolecules (the top is the proposed Y-specific region and the bottom is the rest of LG20_El14.2) and the green boxes represent scaffolds or contigs mapped to this chromosome. Red points represent contacts (close proximity) between regions. There are multiple inversions between the pink salmon and coho salmon genome seen in the dotplot, but the contact map supports the order and orientation for the pink salmon genome assembly and these could represent actual inversions between species instead of assembly errors. (TIF) [file pone.0255752.s005.tif]

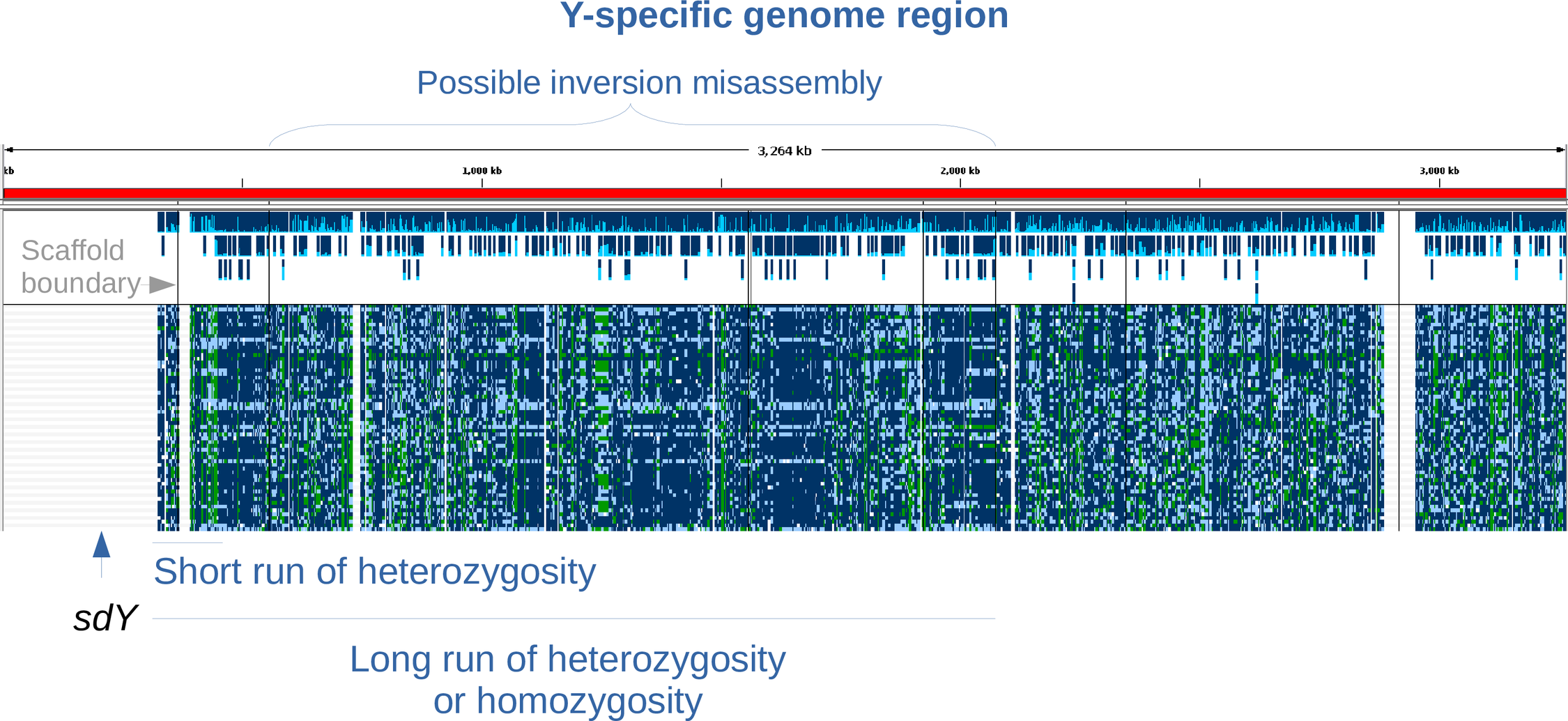

Supplement: S5 Fig — Genotypes are shown from an IGV [112] screenshot for the 61 samples of pink salmon for the region with the sdY sex-determining gene. The top portion shows the distance of the Y-specific genome region (~3.2 Mbp) and the contig/scaffold boundaries that make up this region are shown as vertical lines. Below the distances, allele frequencies for each locus are shown, and below that individual genotypes. The x-axis of the genotypes represent loci and each line on the y-axis represents an individual pink salmon. The dark-blue colour is a homozygous reference genotype, the light-blue colour a heterozygous genotype, and the green genotype is for a homozygous alternative locus. There are large stretches (1–2 Mbp) of heterozygosity and homozygosity based on sex. Please note that there is a possible inversion (from a mis-assembly) in this region as the runs of homozygosity and heterozygosity are broken by a section from ~600 kbp and ~1,300 kbp. (TIF) [file pone.0255752.s006.tif]
